# The role of climatic and geological events in generating diversity in Ethiopian grass frogs (genus *Ptychadena*)

**DOI:** 10.1098/rsos.170021

**Published:** 2017-08-23

**Authors:** Megan L. Smith, Brice P. Noonan, Timothy J. Colston

**Affiliations:** 1 Department of Biology, University of Mississippi, 108 Shoemaker Hall, University, MS 38677, USA; 2 Zoological Natural History Museum, Addis Ababa University, Arat Kilo, Addis Ababa, Ethiopia

**Keywords:** amphibians, coalescent species delimitation, divergence dating, Ethiopia, Ptychadenidae

## Abstract

Ethiopia is a world biodiversity hotspot and harbours levels of biotic endemism unmatched in the Horn of Africa, largely due to topographic—and thus habitat—complexity, which results from a very active geological and climatic history. Among Ethiopian vertebrate fauna, amphibians harbour the highest levels of endemism, making amphibians a compelling system for the exploration of the impacts of Ethiopia's complex abiotic history on biotic diversification. Grass frogs of the genus *Ptychadena* are notably diverse in Ethiopia, where they have undergone an evolutionary radiation. We used molecular data and expanded taxon sampling to test for cryptic diversity and to explore diversification patterns in both the highland radiation and two widespread lowland *Ptychadena*. Species delimitation results support the presence of nine highland species and four lowland species in our dataset, and divergence dating suggests that both geologic events and climatic fluctuations played a complex and confounded role in the diversification of *Ptychadena* in Ethiopia. We rectify the taxonomy of the endemic *P. neumanni* species complex, elevating one formally synonymized name and describing three novel taxa. Finally, we describe two novel lowland *Ptychadena* species that occur in Ethiopia and may be more broadly distributed.

## 1. Introduction

Ethiopia is the cradle of humanity, a hotspot of biodiversity [1] and the second most populated nation in Africa [2]. These factors have led to a confluence of circumstances that have largely obscured the evolutionary history of this unique biotic region. The World Wildlife Foundation has designated the East African Region (Ethiopia–Eritrea) as a Global 200 site, with seven Afrotropical ecoregions, all of which are considered endangered, poorly studied and under critical threat [3]. Covering roughly 1 127 000 km^2^ over three primary geomorphological landscape regions—(i) the northern highlands, (ii) the Rift Valley and (iii) the southern plateau—Ethiopia has a remarkably diverse biota that has evolved amidst a long history of abiotic change [4]. Presently, the Ethiopian highlands range from 1500 to around 3000 m.a.s.l. with depressions as low as −125 m.a.s.l. and are divided by the Great Rift Valley (GRV) into the northwestern and southeastern highlands, each with associated lowlands [4]. Variation in topography and land use in conjunction with seasonal variation in climatic conditions has led to a heterogeneous landscape with a variety of habitats characterized by high levels of endemism, particularly in the highlands [4].

Climatic and geological events are frequently evoked as drivers of diversification, and Ethiopia's history is rife with changes in both climate and topography. For highland taxa, glacial cycles of the Quaternary probably resulted in alternating periods of range expansion—to areas further down mountains and possibly across lowlands—and range contraction—to areas higher up mountains. Such processes have been shown to result in increased diversity and population structure in several taxa [5,6]. Evidence suggests that, within Ethiopia, montane forests expanded during humid periods and were fragmented during more arid periods throughout these Pleistocene climatic changes [7]. Studies have implied diverse effects of Pleistocene climatic changes on Ethiopian flora and fauna including increased connectivity during interglacial periods via ‘montane forest bridges’ (e.g. giant lobelia [7]) and population differentiation due to habitat fragmentation (e.g. Ethiopian wolves [8]). However, climatic changes in Ethiopia were not limited to the Pleistocene, and there have been extreme variations in the availability of moisture in East Africa over time [9]. One well-known example of this variation is East African aridification, which is characterized by the expansion of savannah grasslands into previously wet forests and occurred 8–13.5 Ma [10].

The geological history of Ethiopia is characterized by periods of highland uplift and rift formation. Highland uplift can lead to new niches associated with increased hydrologic and elevational heterogeneity and has been identified as a driver of diversification in taxa such as Neotropical *Pionus* parrots [11]. Rift formation can both act as a dispersal barrier and provide novel habitat (e.g. rift valley lakes) to species. The GRV is part of the East African Rift and runs more than 900 km in Ethiopia between the border with Kenya in the southwest and the border with Djibouti in the northeast [4]. The GRV varies from 50 to 100 km wide and has been shown to be a dispersal barrier for a wide range of taxa, including mammals, amphibians, reptiles, birds and plants [7,8,12–19]. Studies of species spanning the GRV have found results consistent with a role of rift formation and highland uplift in diversification [13,18,19].

Most phylogeographic studies of Ethiopian fauna have been limited to species distributed throughout the highlands, and the effects of the complex landscape on lowland species is relatively unknown (but see [20]). Within Ethiopia, forested lowlands are fragmented by extensive highlands and arid grasslands, creating a patchwork of highly isolated lowlands that may lead to strong population structuring in lowland taxa.

Ethiopian endemics are represented across a wide range of taxa—particularly in charismatic fauna such as birds and mammals [21]. However, Ethiopian amphibians harbour the highest levels of vertebrate endemism, with more than 23 amphibian species endemic to the Ethiopian highlands [22,23]. Among these, the genus *Ptychadena* contains thirteen described species, of which six are endemic [23], making the number of endemic species second only to *Leptopelis*. This high level of diversity and endemism, coupled with the widespread distribution of the genus in both highland and lowland areas of Ethiopia makes this group a compelling system for the study of factors influencing diversification in Ethiopia.

Previous work on highland Ethiopian *Ptychadena* suggested the presence of at least four undescribed species, but these species have not been described to date [12]. A recent study aimed at elucidating the phylogeographic history of several Ethiopian amphibians with distributions spanning the GRV included two described and two undescribed lineages of *Ptychadena* and found evidence of population differentiation on either side of the GRV in two lineages (*P. cooperi* and *P. cf. neumanni 2*) [19]. Freilich *et al*. suggested that elevation and microhabitat differences structure diversity within highland *Ptychadena*, and that the GRV acts as a dispersal barrier for high-elevation species within the genus [12,19].

In this study, our aim is to elucidate how abiotic factors have influenced the diversification of a diverse and widespread group that spans both lowland and highland biomes in Ethiopia. Our objectives are threefold: (i) to explore the phylogenetic relationships of Ethiopian *Ptychadena*; (ii) to resolve species boundaries in this group and (iii) to infer the abiotic factors driving diversification.

## 2. Material and methods

### 2.1. Sampling

**Figure 1.**
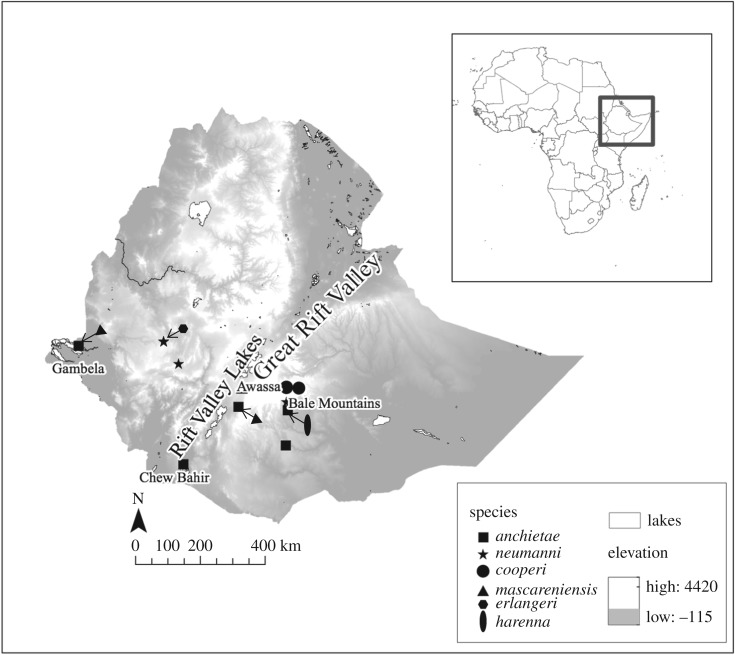
Map showing sampling localities of samples collected by TJC during November 2012–November 2013. Arrows are used to show cases in which more than one species was collected from the same locality. Specimens were originally assigned to species following the morphological identification in Largen & Spawls [23], and those names are given in the symbol legend.

For this study, 54 individuals were collected from 10 localities both within and spanning the GRV (figure 1 and electronic supplementary material, table S1); tissue samples were collected and stored in 95% ethanol. All specimens were deposited at the Zoological Museum of Natural History (ZMNH), Addis Ababa University in Addis Ababa, Ethiopia where they await accessioning. Morphological species identification followed [23] and references therein.

### 2.2. Genetic data and gene tree analyses

Genomic DNA was extracted from muscle or liver tissue using Qiagen DNeasy tissue kits following the manufacturer's standard protocols. We amplified portions of two mitochondrial (12S and 16S) and four nuclear (Tyrosinase, CXCR4, NCX1 and Rag-1) genes. Information on PCR conditions is available in the electronic supplementary material for this article (electronic supplementary material, table S2). Sequencing reactions were performed with BigDye Terminator v. 3.1, and both strands of PCR products were sequenced on an ABI 3730XL automated sequencer following the manufacturer's protocols.

Nucleotide sequences were assembled and aligned using Geneious Pro v. 5.5.8 (Biomatters). An open reading frame for protein coding loci was verified by eye and a search for nonsense mutations was performed. Previously published sequence data for *Ptychadena* and appropriate outgroup species were downloaded from GenBank, and all sequences were aligned using the MUSCLE algorithm in Geneious [24] and edited by eye when necessary. A full list of sequences generated for this study and those acquired from Genbank can be found in the electronic supplementary material for this article (electronic supplementary material, table S1). The best nucleotide substitution model for each gene was determined using PartitionFinder v. 1.1.1 [25], which selects the best-fit partitioning scheme and the best-fit model of molecular evolution simultaneously for nucleotide alignments and has been shown to outperform other approaches, such as *ad hoc* selection of partitioning schemes based on gene or codon position and hierarchical clustering methods [25]. Summary statistics were calculated in MEGA v. 6.06 [26]. Fu and Li's *D*^F^ statistic [27], which uses information about the number of recent mutations to assess neutrality, was calculated in DNAsp v. 5.1.0 [28].

To infer species-level relationships within *Ptychadena* and explore potential cryptic diversity among our samples, we constructed a gene tree using Bayesian Inference (BI) and all available *Ptychadena* 16S sequences from GenBank and sequenced in this study in MrBayes v. 3.2.6 on XSEDE [29] available on the Cipres Science Gateway (http://www.phylo.org/portal2/). Only 16S was used in this analysis in order to maximize our geographical and taxonomic sampling. Analyses were run for 4 000 000 generations over four chains; 4000 trees were sampled, and the first 1000 were discarded as burn-in based on stationarity of the log-likelihood scores. To estimate a more robust hypothesis of species relationships within Ethiopia, BI and maximum-likelihood (ML) methods and the full concatenated mitochondrial and nuclear datasets were used to reconstruct phylogenies for only those samples from Ethiopia (see the electronic supplementary material, figures S1 and S2).

### 2.3. Coalescent species delimitation

To obtain estimates of haplotype diversity and delimit species for the highland taxa, we used three species discovery approaches and one species validation approach. Species discovery approaches require no *a priori* assignment of individuals to putative species, while species validation approaches require that individuals be assigned to putative species. We used two population clustering algorithms (STRUCTURE and Structurama) and one approach aimed at discovering species-level differentiation (spedeSTEM) for species discovery. These approaches were not applied to the lowland taxa, as there were few nuclear sequences available for these taxa outside of Ethiopia; a species delimitation approach requiring only mitochondrial data was used instead (see below). Haplotypes were reconstructed from nuclear data using Phase v. 2.1.1 as implemented in DNAsp v. 5.1.0, with a cut-off of 90% probability [28,30]. STRUCTURE and Structurama analyses used a dataset that included 189 *Ptychadena*, four nuclear loci, and 13% missing data, excluding individuals missing data at more than one locus (with the exception of the two *P. harenna* samples retained to avoid losing all information from this species).

To obtain initial estimates of the number of putative species and the composition of these species, phased nuclear haplotypes were analysed using STRUCTURE v. 2.3.3 [31]. Analyses were run for *K*-values from 2 to 15 with five replicates each, following Freilich *et al.* [12], who ran analyses for *K*-values from 2 to 14. We chose to include *K* = 15, as we included an additional species (*P. harenna*) in our analyses. The first 100 000 generations were discarded as burn-in, and 500 000 generations followed. Result files were then submitted to Structure Harvester to determine the optimal *K*-value [32].

To corroborate STRUCTURE results, nuclear haplotypes were analysed using Structurama [33]. Structurama was run both with the number of populations treated as a random variable and with a set number of populations for *K*-values of 7–9 using the admixture model. All Structurama analyses were run for 100 000 generations, and the first 25% of samples were discarded as burn-in. STRUCTURE and Structurama results were visualized in DISTRUCT v. 1.1.1 [34].

To estimate the number of putative species using ML and AIC model selection methods, nuclear sequences were analysed in spedeSTEM v. 2.0 [35]. SpedeSTEM uses gene trees to estimate the ML species tree in STEM [36] for various models of species delimitation, which are then evaluated using AIC and can be used either for species discovery or for species validation [35,37]. We used spedeSTEM as a species discovery tool and used ML phylogenies generated in RAxML (electronic supplementary material) as the input gene trees.

To test the validity of putative species, we used BP&P3 [38–40]. We assigned individuals to putative species based on the results of Freilich *et al.* [12] and the results of the mtDNA gene trees (see below). A reduced dataset including at least two sequences for each putative species was analysed in BP&P to make this analysis computationally tractable with the gamma priors on population size and root age of *G*(21 000) and *G*(22 000), respectively. We chose a small *α* (*α* = 2), as this represents a diffuse prior, and we had limited prior information on the appropriate value for this parameter. We chose *β* = 1000 to give the distribution on population size a mean of 0.001, a reasonable value for species with small to moderate population sizes. The mean of the prior on divergence times was 0.002, corresponding to 0.002 expected mutations per site, a reasonable value for recently diverged species with small to moderate population sizes. BP&P was run with and without a guide tree on nuclear sequences to test the robustness of results to the provision or lack thereof of a guide tree. We used only nuclear sequences to avoid the circularity associated with using the same data to define putative species and to test the validity of these species.

For the lowland taxa, the ‘splits’ package in R was used to delimit species based on the mitochondrial gene 16S. ‘Splits’ implements the generalized mixed Yule coalescent, which is a likelihood method that fits within- and between-species branching models to reconstructed gene trees [41]. The tree generated in MrBayes (electronic supplementary material) was loaded into R, made ultrametric and analysed using the single threshold model.

### 2.4. Species tree and divergence dating

To account for stochastic differences in the coalescent histories of genes and date diversification events, a chronogram of Ethiopian *Ptychadena* was reconstructed using both mitochondrial and nuclear datasets and the program *BEAST [42] using the BEAGLE framework [43] on XSEDE available on the Cipres Science Gateway (http://www.phylo.org/portal2/). This marks the first use of fossil data to date diversification in Ethiopian *Ptychadena*.

For the *BEAST analysis, we used the same reduced dataset that was analysed in BP&P, but with mitochondrial loci included. Though we excluded the mitochondrial data in the BP&P analysis, we elected to include it in the *BEAST analysis, as populations for this analysis were defined based on the results of all species delimitation and validation analyses. Including the mitochondrial data also makes our results more comparable to those of Freilich *et al.* [12], who used the same loci, but with fewer species and without fossil information to estimate the timing of divergence events. The partitions included 12S, 16S, Tyrosinase, Rag-1, CXCR4 and NCX1. We used the GTR + Gamma model for all partitions. The proportion of invariant sites was not included in the model as associated parameters failed to mix during initial runs [44]. We implemented the relaxed clock model with a lognormal distribution and a calibrated Yule speciation prior. To calibrate a local clock in our analyses, we used the recently described fossils RRBP 07165 and RRBP 05113 to place a constraint on the minimum age of Ptychadenidae at 24.5 Ma [45]. These fossils are isolated compound sacra collected from the Nsungwe Formation (Late Oligocene) in the Rukwa Rift Basin of southwestern Tanzania, which was deposited as a part of a system that developed between 25.5 and 24.5 Ma [45]. In the *BEAST analysis, the formation of the family Ptychadenidae was given a minimum age of 24.5 Ma with a lognormal distribution with a mean of 24.5 and standard deviation of 1.0 and a 95% CI from 24.9 to 43.8 Ma. Analyses were run for 300 000 000 generations, storing every 3000 generations and discarding the first 10% of trees as burn-in. Analyses were repeated six times, 10% of trees were discarded as burn-in for each run and results were combined using LogCombiner v. 2.1.1 [46]. The maximum clade credibility tree was summarized in TreeAnnotator v. 2.2.1 [46], and Tracer v. 1.6 [47] was used to check results for convergence by evaluating ESS values.

## 3. Results

### 3.1. Sampling and gene tree analyses

The concatenated alignment of the two mitochondrial and four nuclear loci resulted in 3444 bases with 873 variable sites and 484 parsimony informative sites. Fu and Li's *D*^F^ statistic was not significant for any of the loci studied, indicating that none of these genes are under selection and that there has been no recent population growth. Summary statistics for each locus are presented in table 1.

**Table 1.** Summary statistics for genes used in this study, where ‘*n*’ is the number of sites, *p*_s_ is the proportion of segregating sites and *π* is nucleotide diversity.

gene	*n*	*p*_s_	*π*	Fu and Li's *D*
12S	442	0.381	0.0700	−1.30
16S	490	0.308	0.0569	1.31
Rag-1	672	0.204	0.0314	0.690
NCX1	990	0.134	0.0200	1.27
Tyrosinase	331	0.245	0.0450	−1.07
CXCR4	121	0.191	0.0222	1.01

**Figure 2.**
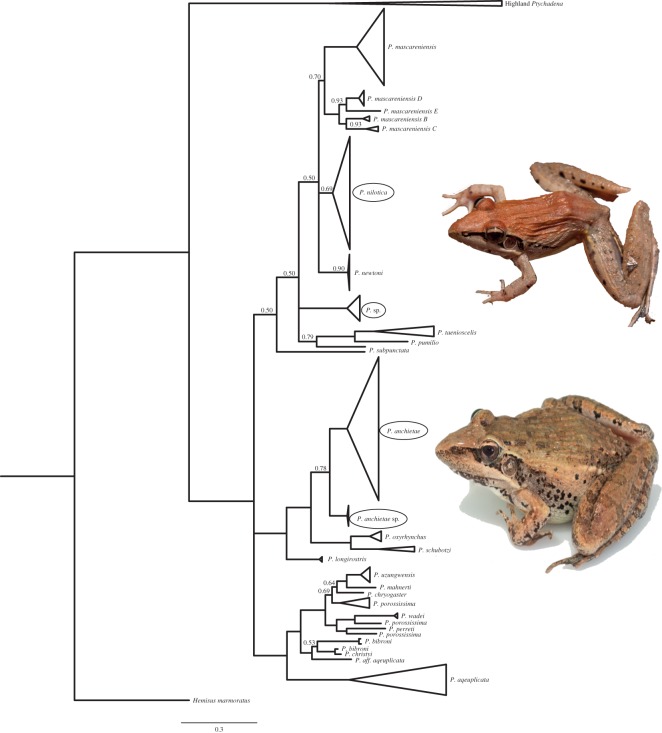
Bayesian gene tree of lowland Ptychadena based on the mitochondrial gene 16S for all samples from GenBank and those collected for this study. Only posterior probabilities less than 0.95 are shown. Circles indicate lowland clades that included samples from Ethiopia collected in this study.

Our combined 16S dataset contains 25 of the 49 described species of *Ptychandena* (ambphibiaweb.org), and 9 of the 13 described *Ptychadena* which occur in Ethiopia. Notably, our dataset included 5 of the 6 described highland-endemic *Ptychadena* in Ethiopia, lacking only *P. wadei*. The Bayesian gene tree based on 16S for all *Ptychadena* lineages supports two of the four lowland lineages sampled here as evolutionarily independent from previously identified lineages (figure 2). Within samples identified as *P. mascareniensis* based on morphology, samples from Lake Awassa within the Rift Valley (figure 1) grouped with *P. nilotica* (Seetzen 1855)*,* which has previously been recorded only in tributaries of the Blue Nile. ‘*P. mascareniensis*’ samples from the Gambela region (figure 1) formed a strongly supported clade with one sample from Tanzania previously identified only as *P.* sp. [48] and belonged to a poorly supported polytomy that included pan-African *P. mascareniensis* lineages (but excluding Ethiopia) as well as *P. pumilio, P. subpunctata* and *P. taenioscelis.* This lineage is referred to as *P.* sp. hereafter.

Within *P. anchietae,* samples from within or east of the GRV belonged to a widespread but poorly supported clade of *P. anchietae* including samples from Kenya, Rwanda, Uganda, the Democratic Republic of the Congo, Somalia and South Africa*.* However, samples identified as *P. anchietae* collected from the Gambela region (figure 1) formed a highly supported clade sister to all other *P. anchietae*, and will be referred to as *P. cf. anchietae Gambela* hereafter*.*

Bayesian and ML trees (electronic supplementary material, figure S1) recovered seven of the eight highland lineages suggested by Freilich *et al.* [12]—*P. cf. neumanni* 1–5*, P. cooperi and P. nana—*as well as one corresponding to *P. erlangeri* that also contained the previously unsampled species *P. harenna.*

### 3.2. Coalescent species delimitation

Structure Harvester results supported seven clusters (ln = −2982.0000, delta-*K* = 223.112428). STRUCTURE results for *K*-values of 8 and 9 are also discussed, as 8 or 9 species were supported by mitochondrial clades (electronic supplementary material, figure S1) and by Structurama (see below). Structurama recovers either 8 or 9 as the optimal number of species, depending on the shape and scale of the gamma distribution specified for the number of clusters. Structurama was also run using the admixture model with *K* set to 7 (based on Structure Harvester results), 8 and 9.

**Figure 3.**
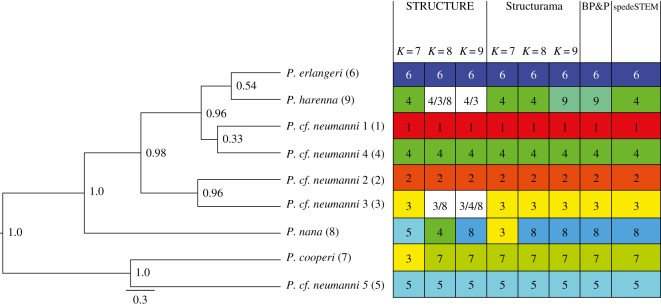
Species tree generated in *BEAST with posterior probabilities. To the right, numbers indicate population (STRUCTURE and Structurama) or species (BP&P and spedeSTEM) affiliation of the taxa. Numbers to the right of the taxa names correspond to the numbers inside the blocks indicating species/populations recovered in each analysis.

**Figure 4.**
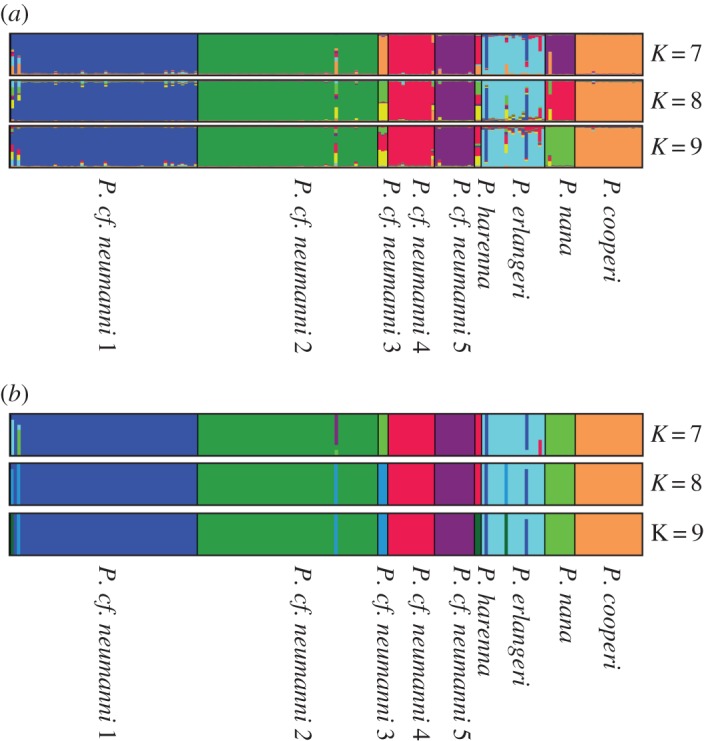
Visualization of the Bayesian clustering analysis (*a*) from the program STRUCTURE for *K*-values 7, 8 and 9, with membership coefficients displayed on the *y*-axis and individuals grouped along the *x*-axis based on mt clade membership and (*b*) from the program Structurama for *K*-values of 7, 8 and 9. Graphs were generated in DISTRUCT v. 1.1 [34].

All STRUCTURE and Structurama results (figures 3 and 4) support the distinctness of *P. cf. neumanni* 1, *P. cf. neumanni* 2 and *P. erlangeri. Ptychadena cf. neumanni* 5 and *P. cooperi* were supported as distinct clusters when *K* was set to 8 or 9 in STRUCTURE and at all three *K*-values in Structurama. *Ptychadena nana* was recovered as a distinct cluster in STRUCTURE when *K* was set to 9 and in Structurama when *K* was set to 8 or 9. *Ptychadena cf. neumanni 3* was recovered as a distinct cluster in Structurama at *K*-values of 8 and 9. *Ptychadena cf. neumanni 4* was recovered as a distinct cluster at *K*-values of 7 and 8 in STRUCTURE and at *K* = 9 in Structurama. *Ptychadena harenna* was recovered as a distinct cluster in Structurama when *K* was set to 9 but was clustered with *P. cf. neumanni* 4 at *K* = 7 or 8.

In spedeSTEM, the model with the highest model probability (*ω* = 1.000000) included eight species, with all lineages corresponding to those recovered in the Structurama analysis when *K* was set to 8 (figure 3). *P. cf. neumanni* 4 and *P. harenna* were not supported as separate species by spedeSTEM.

In BP&P, we tested the validity of the nine species identified by Structurama at *K* = 9. BP&P supported the validity of all nine highland species with a posterior probability of 0.90 (figure 3). All species had posterior probabilities greater than 0.99 except for *P. cf. neumanni 4* and *P. harenna*, which both had a posterior probability of 0.90. Results did not differ when BP&P was run with or without a guide tree. These results corroborate those of Freilich *et al.* [12] in highlighting the distinctiveness of highland *Ptychadena* in Ethiopia.

The ‘splits’ package recovered 13 lowland species, including *P.* sp., but not *P. cf. anchietae Gambela.* However, when *P. anchietae* and *P. cf. anchietae Gambela* were analysed separately from other lowland species, *P. cf. anchietae Gambela* was recovered as a distinct species.

### 3.3. Species tree analysis

**Figure 5.**
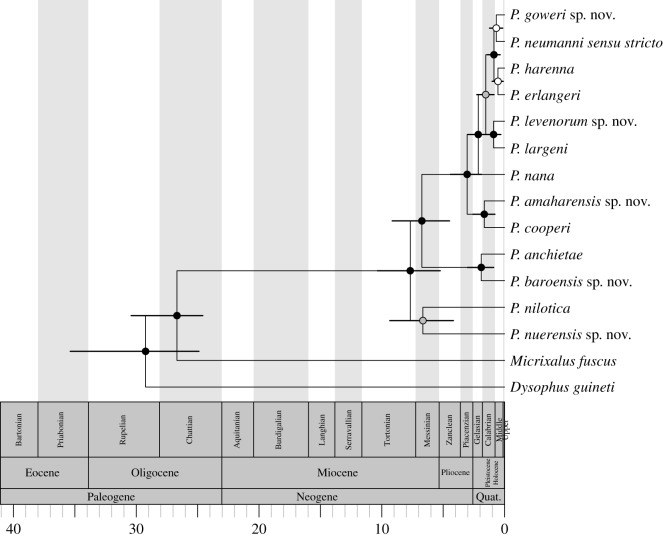
Original species-tree chronogram generated in *BEAST for Ethiopian *Ptychadena*. Bars represent 95% CI. Taxa names correspond to the revised taxonomy in this study.

The species tree generated in *BEAST (figures 3 and 5) recovered all highland species as monophyletic, and recovered *P. nilotica* and *P. sp*. as sister to all other Ethiopian *Ptychadena*, and *P. anchietae* and *P. cf. anchietae Gambela* as distinct and sister to the highland clade. The time-calibrated species tree indicates that the origin of *Ptychadena* occurred in the Oligocene (26.7 Ma, 95% CI: 24.6–30.4 Ma). The species tree indicates that the radiation of the highland lineages began in the Pliocene (3.0 Ma, 95% CI: 1.9–4.4 Ma), later than suggested in previous studies [12], and continued through the Pleistocene with the most recent divergent event occurring 0.54 Ma (95% CI: 0.1–1.0 Ma) between *P. erlangeri* and *P. harenna*, whereas diversification of the lowland lineages is estimated to have occurred during the Pliocene.

## 4. Discussion

### 4.1. Coalescent species delimitation and taxonomy

#### 4.1.1. Cryptic diversity in Ethiopian *Ptychadena*

Several species of *Ptychadena* in Ethiopia have been suggested to be likely complexes of closely related morphologically cryptic species including *P. anchietae*, *P. mascareniensis* and *P. neumanni* [12,23,49]. With increased individual, population and taxonomic sampling of Ethiopian *Ptychadena* compared to previous studies and by using multiple, independent loci and coalescent species discovery and delimitation approaches, we were able to accurately assess intraspecific cryptic diversity and the timing of diversification events within lowland and highland lineages in Ethiopia. Our results suggest that taxonomic revisions are necessary, and we discuss those below*.* Lowland Ethiopian lineages collected for this study consist of two lineages that appear to be evolutionarily independent from all currently recognized lowland *Ptychadena* species and two lineages that are members of two widespread species.

Samples morphologically identified as *P. anchietae* and *P. mascareniensis* that grouped with more widespread lineages (*P. anchietae* and *P. nilotica,* respectively) were collected from localities within the Great Rift Valley including locations near Awassa and Chew Bahir (figure 1). Our results highlight that *P. nilotica*, a species that is morphologically similar to *P. mascareniensis*, inhabits the Rift Valley lakes. It is likely that individuals previously described as *P. mascareniensis* that inhabit the GRV and northwards through the valleys associated with the Blue Nile River are in fact *P. nilotica* and that this species is widespread in Ethiopia, whereas *P. mascareniensis sensu stricto* may not be present in Ethiopia. The widespread *P. anchietae* includes samples from Kenya, Rwanda, Uganda, the Democratic Republic of the Congo, Somalia and South Africa, in addition to samples from Ethiopia. Our results consistently support that *P. cf. anchietae Gambela* is an independent lineage and should be given full species status.

Additionally, *Ptychadena*. sp. was recovered as an independent lineage from the widespread *P. mascareniensis* in all analyses and should also be elevated. Interestingly, *P. cf. anchietae Gambela* and *P.* sp*.* appear to overlap, having been collected from the same locality on the Baro River (a tributary to the White Nile) in the Gambela region, which is located within the Baro-Akoba river basin (figure 1). This habitat is markedly different from that of the Rift Valley lakes and other tributaries of the Blue Nile in northwestern Ethiopia. We give a full description of both of these novel species below.

Our results corroborate those of Freilich *et al.* [12] in highlighting the need for taxonomic revisions for the highland *Ptychadena* in Ethiopia. Our results support the existence of nine distinct highland species, plus *P. wadei* which was not in our dataset, bringing the total number of highland-endemic *Ptychadena* in Ethiopia to 10. The species complex currently recognized as *P. neumanni* is a paraphyletic group consisting of six distinct evolutionary lineages (*P. cf. neumanni* 1–5 and *P. harenna*)*.* These lineages were recovered as distinct in all clustering analyses excluding spedeSTEM, and were retained as independent lineages in our BP&P analysis. The other, more morphologically distinct, highland species—*P. cooperi, P. erlangeri* and *P. nana*—were also recovered as evolutionary independent lineages in all analyses. Therefore, we propose that there are at least ten distinct highland *Ptychadena* species in Ethiopia, including three novel species we describe below. We do not find evidence that the divergent populations across the GRV in *P. cooperi* and *P. cf. neumanni 2* uncovered by Freilich *et al*. [19] warrant elevation to species status based on the data collected here.

#### 4.1.2. Taxonomy

The coalescent species discovery and delimitation tools we employed are strict in their methods of assigning independent evolutionary units, and we feel that the four lowland species and nine highland species models we recovered are conservative. These methods are becoming more widespread in their use to delimit species when morphological identification can be tenuous (e.g. [50,51]) and DNA-based species diagnosis has been recommended to be included in all formal *Codes* [52]. Additionally, nearly all novel independent lineages we recovered appear to be allopatric; with highland species stratified by elevation or when found in the same locality can be distinguished by their ecology or adult morphology [12] (table 2). Given that all amphibian taxa are under threat due to climate change, and the endemic nature of these Ethiopian *Ptychadena,* we feel that not only is this taxonomic revision strongly supported, but also that it is imperative to conservation efforts. Below we update the taxonomy of *Ptychadena* in Ethiopia with one resurrected name and give descriptions for five novel species. All type specimens are designated by their field collection tag numbers until accession numbers become available from the ZMNH where they await accessioning. Representative photos from all species can be found in the supplemental information of this paper or supplemental material of [12].

**Table 2.** Habitat, altitudinal range and distribution of Ethiopian highland-endemic *Ptychadena.*

species	habitat	distribution in Ethiopia	altitudinal range (m)
*P. amarensis*	montane streams and grasslands	Highlands west of the GRV	2403–2532
*P. cooperi*	streams	Highlands on both sides of GRV	2387–3045
*P. erlangeri*	clearings in moist evergreen forests	Highlands west of the GRV	1409–2322
*P. goweri*	streams in moist evergreen forests	Highlands east of the GRV	2322–2550
*P. largeni*	clearings and montane grasslands	Highlands on both sides of GRV	2541–3087
*P. levenorum*	montane grasslands	Endemic to the Bale Mountains	2326–3023
*P. nana*	montane grasslands	Highlands east of the GRV	2584–3023
*P. neumanni*	clearings in moist evergreen forests	Highlands west of the GRV	1695–2468

i. Lowland taxa

*Ptychadena baroensis* **sp. nov**

*Holotype:* Timothy J. Colston (TJC) 318, Telouse, Lare, Gambela Region, Ethiopia, 8.26557° N, 33.94688° E, 414 m; collected by Timothy J. Colston on 18 April 2013. Paratypes = TJC319, TJC343–344.
*Diagnosis:* This species includes all populations and individuals that cluster with *P. cf.* sp. Gambela 1 used in this study with strong support in the coalescent species model. This species can be distinguished from all other similar or related taxa by the following characters in combination: *P. baroensis* is a moderately large *Ptychadena* with males known to reach an s.v.l. of 47.8 mm. The dorsum is brown to tannish red, may have spots and is covered by a series of longitudinal skin ridges that are often indistinct or broken. The triangular patch on the snout is less pronounced than that of *P. anchieta sensu stricto*, which in Ethiopia is only known from lowlands east of the GRV. Webbing on the hind feet is extensive with usually only two phalanges free on the longest toe. The back of the thigh is often marked with yellow and black longitudinal bands and the ventral side of the body is white to pale yellow.
*Distribution: P. baroensis* is known to occur in humid grasslands and marshes near permanent water, particularly tributaries of the White Nile, in lowlands west of the GRV in Ethiopia; but may be more widespread into neighbouring countries.
*Etymology:* This species is named after the Baro River, a tributary of the White Nile, on the banks of which the type specimen was collected.

*Ptychadena nuerensis* **sp. nov.**

*Holotype:* TJC455, Telouse, Lare, Gambela Region, Ethiopia, 8.26875° N, 33.94616° E, 415 m; collected by Timothy J. Colston on 6 May 3013. Paratypes = TJC410, TJC451.
*Diagnosis:* This species includes all populations and individuals that cluster with *P.* sp. used in this study with strong support in the coalescent species model. This species can be distinguished from all other similar or related taxa by the following characters in combination: *P. nuerensis* is a moderately large *Ptychadena* with males known to reach an s.v.l. of 41.6 mm. The dorsum is brown to brick red, may have spots, usually possesses a cream or yellow vertebral band and is covered by a series of longitudinal skin ridges that are often indistinct or broken. Webbing on the hind feet is moderate with at least two to three phalanges free on the longest toe. The back of the thigh is mottled and marked with yellow and black longitudinal bands and the ventral side of the body is white to pale yellow. The dorsal side of the thighs is typically boldly marked with dark crossbars.
*Distribution: P. nuerensis* is known to occupy lowland savannahs, particularly at the margins of permanent water, west of the GRV in Ethiopia; but may be more widespread into neighbouring countries.
*Etymology:* This species is named in honour of the Nuer Tribe of South Sudan and western Ethiopia, including the type locality of Telouse, Gambela, for their enthusiasm and support of TJC during his fieldwork in their lands.

ii. Highland taxa—*Ptychadena neumanni* species complex. These taxa are a group of highly morphologically variable and similar species that can be identified in the field according to their distribution and ecology (table 2).

*Ptycandena neumanni sensu stricto* (Ahl 1924)

*Rana* (*Ptychadena*) *neumanni* Ahl 1924, Perret 1980
*Diagnosis:* This species includes all populations and individuals that cluster with *P. cf. neumanni* 1 used in this study with strong support in the coalescent species model. This species can be distinguished from all other similar or related taxa by the following characters in combination: *P. neumanni* is a moderate-sized frog with males known to reach an s.v.l. of 39 mm. The dorsum is tan to olive greenish, often with a vertebral band or thin line that may be cream, yellow, green, orange or tan. Several longitudinal ridges that may be broken run the length of the dorsum, which may have dark blotches. The flanks often possess red or orange pigment and the underside is cream to white.
*Distribution: P. neumanni* is known to occupy an altitudinal range of 1500–2500 m.a.s.l. in Ethiopian highlands west of the GRV. Individuals used in this study include the elevation range and distribution of their type locality: Gadat, Gofa, Ethiopia (as restricted by PERRET 1980: 157).

*Ptychadena largeni* (Perret 1994)

*Ptychadena largeni* Perret 1994
*Ptychadena neumanni* Largen 2001
*Diagnosis:* This species includes all populations and individuals that cluster with *P. cf. neumanni* 2 used in this study with strong support in the coalescent species model. This species can be distinguished from all other similar or related taxa by the following characters in combination: *P. largeni* is a medium-sized frog with males known to reach an s.v.l. of 32 mm. The dorsum is tan to olive greenish, often with a vertebral band or more often a thin line that may be cream, yellow, green or tan. Several longitudinal ridges that may be broken run the length of the dorsum and these ridges may be replaced by tubercles. The underside is cream to white.
*Distribution: P. largeni* is known to occupy an altitudinal range of 2500–3100 m.a.s.l. in Ethiopian highlands on both sides of the GRV. Individuals used in this study include the elevation range and distribution of their type locality: Addis Ababa, Shewa (Shoa) 09°02′ N 38°45′ E, 2500 m.a.s.l.

*Ptychadena levenorum* **sp. nov.**

*Holotype:* TJC219, Katcha, Bale Mountains National Park, Ethiopia, 6.71645° N, 39.72484° E, 2326 m; collected by Timothy J. Colston on 8 December 2012. Paratypes = Xenia Freilich (XF) 923, XF927.
*Diagnosis:* This species includes all populations and individuals that cluster with *P. cf. neumanni* 3 used in this study with strong support in the coalescent species model. This species can be distinguished from all other similar or related taxa by the following characters in combination: *P. levenorum* is a medium-sized frog with males known to reach an s.v.l. of 31.9 mm. The dorsum is tan to olive green, often with a vertebral band or thin line that may be cream, yellow, green or tan. Several longitudinal ridges that may be broken run the length of the dorsum, which may have dark blotches. The thighs possess dark crossbars and usually have a tibial stripe.
*Distribution: P. levenorum* is known to occupy an altitudinal range of 2300–3100 m.a.s.l. in the Bale Mountains of southern Ethiopia.
*Etymology:* This species is named in honour of Guy and Yvonne Levene of the Bale Mountains Lodge for their numerous and continued contributions to conservation efforts within Ethiopia, most notably within the Bale Mountains National Park.

Ptychadena goweri **sp. nov**.

*Holotype:* TJC224, Katcha, Bale Mountains National Park, Ethiopia, 6.71779° N, 39.72572° E, 2375 m; collected by Timothy J. Colston on 10 December 2012. Paratypes = XF781–783.
*Diagnosis:* This species includes all populations and individuals that cluster with *P. cf. neumanni* 4 used in this study with strong support in the coalescent species model. This species can be distinguished from all other similar or related taxa by the following characters in combination: *P. goweri* is a medium-sized frog with females known to reach an s.v.l. of 33.7 mm. The dorsum is tan to olive green, often with a vertebral band or thin line that may be cream, yellow, green or tan. The flanks are suffused with orange–red or yellow pigment and black or green mottles. The underside is white.
*Distribution: P. goweri* is known to occupy an altitudinal range of 2300–2600 m.a.s.l. in Ethiopian highland streams east of the GRV.
*Etymology:* This species is named in honour of herpetologist Dr David Gower for his contributions to systematics and taxonomy of East African amphibians, and his conservation efforts in Ethiopia.

*Ptychadena amharensis* **sp. nov.**

*Holotype:* XF140, Dejen, Amhara Region, Ethiopia; 10.190778° N, 38.140073° E; collected by Xenia Freilich. Paratypes = XF141–143.
*Diganosis:* This species includes all populations and individuals that cluster with *P. cf. neumanni* 5 used in this study with strong support in the coalescent species model. This species can be distinguished from all other similar or related taxa by the following characters in combination: *P. amharensis* males and females can attain an s.v.l. of 40 mm and 51 mm, respectively. The dorsum may be tan to olive green, mottled with black splotches and may possess a yellow or cream vertebral stripe. The underside is white or cream. The flanks typically possess yellow pigment with tan or brown mottles.
*Distribution: P. amharensis* is known to occupy an altitudinal range of 2400–2600 m.a.s.l. in Ethiopian highlands west of the GRV.
*Etymology:* This species is named after the Amhara region of Ethiopia where the type locality is found.

### 4.2. Abiotic factors influencing diversification

#### 4.2.1. Pleistocene glacial cycles

Much of the diversification within highland lineages is estimated to have occurred during the Pleistocene, with the most recent divergence event having occurred approximately 450 000 years ago. For the past 700 000 years, there have been 100 000 year cycles of glacial and interglacial periods, and the timing of these Pleistocene glacial cycles overlaps with the 95% CI for diversification between *P. erlangeri, P. goweri, P. harenna* and *P. neumanni* (figure 5)*.* This adds to a growing body of evidence for Pleistocene glacial cycles as drivers of diversification [5,6] and corroborates existing evidence of the effects of these cycles on Ethiopian flora and fauna [7,8].

#### 4.2.2. East African aridification

The lowland lineages *P. nilotica* and *P. nuerensis* are estimated to have diverged from each other 6.7 Ma (4.2–9.3 Ma). The timing of this diversification is consistent with East African aridification (8–13.5 Ma, [9], which was characterized by the expansion of savannah grasslands into previously wet forests. This aridification may have resulted in decreased dispersal ability for these lowland amphibians, which have limited protection against desiccation and would probably have experienced habitat reduction and fragmentation during dry periods. Aridification events are supported as drivers of population fragmentation and divergence in several amphibian taxa including the common toad in Central Asian deserts [53], tailed frogs in the Pacific Northwest of the USA [54] and Australian toadlets [55]. That the diversification between *P. nilotica* and *P. nuerensis* is temporally coincident with aridification lends support to this as a driving force in the diversification of lowland Ethiopian *Ptychadena*.

#### 4.2.3. Rift propagation

Extreme southward rift propagation is estimated to have occurred between 8 and 5 Ma in Ethiopia and to have continued until 3 Ma [56]. This timing overlaps with the beginning of the highland radiation (1.9–4.4 Ma) and with diversification between *P. nilotica* and *P. nuerensis* (4.2–9.3 Ma). Rift formation has been shown to correspond to diversification events in other Ethiopian taxa including African clawed frogs [13] and African spitting cobras [18]. It is more difficult to interpret the correspondence of highland diversification in *Ptychadena* and rift formation, as sister species pairs which occur on opposite sides of the GRV (e.g. *P. goweri* and *P. neumanni*) have only recently diverged, and some species (e.g. *P. cooperi* and *P. largeni*) have distributions spanning the GRV. It is feasible that gene flow between lineages of species spanning the GRV occurred at times when the intervening habitat was more hospitable than it is currently, such as during Pleistocene glacial cycles, and that lineages have recently become more isolated and will continue to differentiate if intervening habitat remains inhospitable. For example, we might expect to see diversification within *P. cooperi* on either side of the GRV in the future. In fact, Freilich *et al.* [19] found evidence of differentiation of populations on either side of the GRV in *P. cooperi.* It is also likely that rift formation affected the climate of Ethiopia substantially, and thus rift formation could have been a driver of diversification even in taxa with distributions restricted to one side of the valley.

#### 4.2.4. Highland uplift

Highland uplift is estimated to have occurred in Ethiopia from the Mid–Late Miocene to 2 Ma, and therefore probably played an important role in the diversification of Ethiopian *Ptychadena*. Most notably, the radiation of highland lineages in Ethiopia corresponds to this same time period of highland uplift, approximately 3.0 Ma (95% CI: 1.9–4.4 Ma). Uplift would have resulted in new niches at higher elevations becoming available, and the radiation of highland Ethiopian *Ptychadena* may have been spurred by this new niche availability. Freilich *et al*. [12] highlighted the elevational distributions of these highland taxa, pointing out that *P. erlangeri*, *P. goweri* and *P. neumanni* occupy habitats at elevations below 2500 m, whereas other highland lineages occur at higher elevations. Our findings also suggest altitudinal stratification within highland *Ptychadena*, with all members of the clade containing *P. erlangeri*, *P. goweri, P. harenna* and *P. neumanni* occurring at elevations below 2700 m. All other highland *Ptychadena* were collected exclusively from elevations above 2400 m. This phylogenetic clustering of taxa with similar altitudinal distributions supports a potential role of highland uplift in lineage diversification.

Diversification between lowland *P. anchietae* and *P. baroensis* also occurred during times of highland uplift (95% CI: 0.9–3.0 Ma). Highland uplift may have contributed to the isolation of *P. baroensis*, which appears localized to the Gambela region (or at least tributaries of the White Nile) in Ethiopia, but probably ranges in neighbouring South Sudan (figure 1), from *P. anchietae* populations throughout the rest of sub-Saharan Africa. Further sampling within both Ethiopia and neighbouring countries will be necessary to fully understand the distribution of *P. baroensis*.

### 4.3. Conclusions and future directions

Our objective was to elucidate how abiotic factors have influenced biotic diversification of Ethiopian *Ptychadena*. Our results highlight the presence of undescribed diversity within the widespread lowland taxa, and support and expand the cryptic diversity of highland Ethiopian *Ptychadena* reported by Freilich *et al.* [12]. Our analysis of the timing of *Ptychadena* diversification highlights the importance of several climatic and geologic events in the diversification of Ethiopian *Ptychadena* and corroborates the role of Pleistocene glacial cycles in the diversification of highland species. Future studies should seek to include detailed natural history data on these species, including descriptions of breeding and calling characteristics coupled with molecular-based species identification.

## Supplementary Material

Supporting Information for "The Role of Climatic and Geological Events in Generating Diversity in Ethiopian Grass Frogs (Genus Ptychadena)

## Data accessibility

Alignments have been uploaded to the Dryad Digital Repository (http://dx.doi.org/10.5061/dryad.2s19v) [58]. Sequence data have been deposited in GenBank under Accession numbers MF078078-MF078331.
