# Correction to ‘The role of climatic and geological events in generating diversity in Ethiopian grass frogs (genus *Ptychadena*)’

**DOI:** 10.1098/rsos.171389

**Published:** 2017-10-18

**Authors:** Megan L. Smith, Brice P. Noonan, Timothy J. Colston

## 1. Introduction

This correction is to fulfil the requirements of the International Commission on Zoological Nomenclature (ICZN) code criterion for the online-only publication of a new species name. In order for the five described *Ptychadena* species to be valid, the ICZN requires that the publication containing the species description be registered on ZooBank at the time of publication and that the ZooBank number appear in the publication, along with the species description. Below, we give the ZooBank LSID number for the article and for each of the described species, along with the taxonomic section of the original article.

## 2. ZooBank LSIDs

Original publication (Smith *et al.* [1])

urn:lsid:zoobank.org:pub:70FE6182-CDF1-4C70-A275-54BE27 27B4E4

Correction

urn:lsid:zoobank.org:pub:A36FA32C-19F8-491D-B2CE-BD13 B388AC93

New species: *Ptychadena baroensis* **sp. nov.**

urn:lsid:zoobank.org:act:BBA49EF1-59D1-444E-AA63-B6A8FE825A46

New species: *Ptychadena nuerensis* **sp. nov.**

urn:lsid:zoobank.org: act:966FA5D5-A070-46F2-BD09-246949037A6A

New species: *Ptychadena levenorum* **sp. nov.**

urn:lsid:zoobank.org: act:C4217C69-9935-459D-B545-17525ED6E17F

New species: *Ptychadena goweri* **sp. nov***.*

urn:lsid:zoobank.org: act:37FA41E9-7298-427B-A895-55422E6D3331

New species: *Ptychadena amharensis* **sp. nov.**

urn:lsid:zoobank.org: act:CD1DD194-3433-4ECC-94E8-E5290C1710A2

## 3. Systematics

(i) Lowland taxa

*Ptychadena baroensis* **sp. nov.**

*Holotype:* Timothy J. Colston (TJC) 318, Telouse, Lare, Gambela Region, Ethiopia, 8.26557° N, 33.94688° E, 414 m; collected by Timothy J. Colston on 18 April 2013. Paratypes = TJC319, TJC343-344.

*Diagnosis:* This species includes all populations and individuals that cluster with *P.cf.* sp. Gambela 1 used in this study with strong support in the coalescent species model. This species can be distinguished from all other similar or related taxa by the following characters in combination: *P. baroensis* is a moderately large *Ptychadena* with males known to reach an s.v.l. of 47.8 mm. The dorsum is brown to tannish red, may have spots and is covered by a series of longitudinal skin ridges that are often indistinct or broken. The triangular patch on the snout is less pronounced than that of *P. anchieta sensu stricto*, which in Ethiopia is only known from lowlands east of the GRV. Webbing on the hind feet is extensive with usually only two phalanges free on the longest toe. The back of the thigh is often marked with yellow and black longitudinal bands and the ventral side of the body is white to pale yellow.

*Distribution: Ptychadena baroensis* is known to occur in humid grasslands and marshes near permanent water, particularly tributaries of the White Nile, in lowlands west of the GRV in Ethiopia; but may be more widespread into neighbouring countries.

*Etymology*: This species is named after the Baro River, a tributary of the White Nile, on the banks of which the type specimen was collected.

*Ptychadena nuerensis* **sp. nov.**

*Holotype:* TJC455, Telouse, Lare, Gambela Region, Ethiopia, 8.26875° N, 33.94616° E, 415 m; collected by Timothy J. Colston on 6 May 2013. Paratypes = TJC410, TJC451.

*Diagnosis:* This species includes all populations and individuals that cluster with *P.sp.* used in this study with strong support in the coalescent species model. This species can be distinguished from all other similar or related taxa by the following characters in combination: *P. nuerensis* is a moderately large *Ptychadena* with males known to reach an s.v.l. of 41.6 mm. The dorsum is brown to brick red, may have spots, usually possesses a cream or yellow vertebral band and is covered by a series of longitudinal skin ridges that are often indistinct or broken. Webbing on the hind feet is moderate with at least two to three phalanges free on the longest toe. The back of the thigh is mottled and marked with yellow and black longitudinal bands and the ventral side of the body is white to pale yellow. The dorsal side of the thighs is typically boldly marked with dark crossbars.

*Distribution: Ptychadena nuerensis* is known to occupy lowland savannahs, particularly at the margins of permanent water, west of the GRV in Ethiopia; but may be more widespread into neighbouring countries.

*Etymology:* This species is named in honour of the Nuer Tribe of South Sudan and western Ethiopia, including the type locality of Telouse, Gambela, for their enthusiasm and support of TJC during his fieldwork in their lands.

(ii) Highland taxa—*Ptychadena neumanni* species complex. These taxa are a group of highly morphologically variable and similar species that can be identified in the field according to their distribution and ecology ([1], Table 2).

*Ptychadena levenorum* **sp. nov.**

*Holotype:* TJC219, Katcha, Bale Mountains National Park, Ethiopia, 6.71645° N, 39.72484° E, 2326 m; collected by Timothy J. Colston on 8 December 2012. Paratypes = Xenia Freilich (XF) 923, XF927.

*Diagnosis:* This species includes all populations and individuals that cluster with *P.cf. neumanni* 3 used in this study with strong support in the coalescent species model. This species can be distinguished from all other similar or related taxa by the following characters in combination: *P. levenorum* is a medium-sized frog with males known to reach an s.v.l. of 31.9 mm. The dorsum is tan to olive greenish, often with a vertebral band or thin line that may be cream, yellow, green or tan. Several longitudinal ridges that may be broken run the length of the dorsum, which may have dark blotches. The thighs possess dark crossbars and usually have a tibial stripe.

*Distribution: Ptychadena levenorum* is known to occupy an altitudinal range of 2300–3100 m.a.s.l. in the Bale Mountains of southern Ethiopia.

*Etymology:* This species is named in honour of Guy and Yvonne Levene of the Bale Mountains Lodge for their numerous and continued contributions to conservation efforts within Ethiopia, most notably within the Bale Mountains National Park.

*Ptychadena goweri* **sp. nov.**

*Holotype:* TJC224, Katcha, Bale Mountains National Park, Ethiopia, 6.71779° N, 39.72572° E, 2375 m; collected by Timothy J. Colston on 10 December 2012. Paratypes = XF781–83.

*Diagnosis:* This species includes all populations and individuals that cluster with *P.cf. neumanni* 4 used in this study with strong support in the coalescent species model. This species can be distinguished from all other similar or related taxa by the following characters in combination: *Ptychadena goweri* is a medium-sized frog with females known to reach an s.v.l. of 33.7. The dorsum is tan to olive greenish, often with a vertebral band or thin line that may be cream, yellow, green or tan. The flanks are suffused with orange-red or yellow pigment and black or green mottling. The underside is white.

*Distribution: Ptychadena goweri* is known to occupy an altitudinal range of 2300–2600 m.a.s.l. in Ethiopian highland streams east of the GRV.

*Etymology:* This species is named in honour of herpetologist Dr David Gower for his contributions to systematics and taxonomy of East African amphibians, and his conservation efforts in Ethiopia.

*Ptychadena amharensis* **sp. nov.**

*Holotype:* XF140, Dejen, Amhara Region, Ethiopia; 10.190778° N, 38.140073° E; collected by Xenia Freilich. Paratypes = XF141–143.

*Diganosis:* This species includes all populations and individuals that cluster with *P.cf. neumanni* 5 used in this study with strong support in the coalescent species model. This species can be distinguished from all other similar or related taxa by the following characters in combination: *P. amharensis* males and females can attain an s.v.l. of 40 mm and 51 mm, respectively. The dorsum may be tan to olive green, mottled with black splotches and may possess a yellow or cream vertebral stripe. The underside is white or cream. The flanks typically possess yellow pigment with tan or brown mottles.

*Distribution: Ptychadena amharensis* is known to occupy an altitudinal range of 2400–2600 m.a.s.l. in Ethiopian highlands west of the GRV.

*Etymology:* This species is named after the Amhara region of Ethiopia where the type locality is found.
