# Supplementary material for: The role of climatic and geological events in generating diversity in Ethiopian grass frogs (genus Ptychadena)
Source: R Soc Open Sci. 2017 Aug 23;4(8):170021. doi: 10.1098/rsos.170021 (PMC5579079; doi:10.1098/rsos.170021)
Supplement: Supporting Information for "The Role of Climatic and Geological Events in Generating Diversity in Ethiopian Grass Frogs (Genus Ptychadena) [file rsos170021supp1.pdf]

Table S1: Samples Used in This Study with GenBank Accession Numbers

Specimen number	Species	Source	16S	12S	CXCR4	NCX1	Rag-1	Tyr	GPS coordinates	Country of Origin
XF-136	P. neumanni 1	Freilich et al. (2014)	KF380293	NA	KF379914	KF379840	NA	KF380108	N10° 1.3911' E38° 14.7402'	Ethiopia
XF-137	P. neumanni 1	Freilich et al. (2014)	KF380294	NA	KF379915	KF379841	NA	KF380109	N10° 1.3911' E38° 14.7402'	Ethiopia
XF-138	P. neumanni 1	Freilich et al. (2014)	NA	NA	KF379916	KF379842	KF380651	NA	N10° 1.3911' E38° 14.7402'	Ethiopia
XF-139	P. neumanni 1	Freilich et al. (2014)	KF380295	NA	KF379917	KF379843	KF380652	KF380110	N10° 1.3911' E38° 14.7402'	Ethiopia
XF-196	P. neumanni 1	Freilich et al. (2014)	KF380309	NA	KF379935	KF379844	NA	KF380124	N8° 52.6927' E38° 47.1347'	Ethiopia
XF-200	P. neumanni 1	Freilich et al. (2014)	KF380310	NA	NA	KF379845	NA	KF380125	N8° 57.9585' E38° 47.4199'	Ethiopia
XF-202	P. neumanni 1	Freilich et al. (2014)	NA	NA	NA	NA	NA	NA	N8° 57.9585' E38° 47.4199'	Ethiopia
XF-213	P. neumanni 1	Freilich et al. (2014)	KF380311	NA	KF379936	KF379846	NA	KF380126	N8° 51.3351' E38° 48.1215'	Ethiopia
XF-214	P. neumanni 1	Freilich et al. (2014)	KF380312	NA	KF379937	KF379847	NA	KF380127	N8° 51.3351' E38° 48.1215'	Ethiopia
XF-215	P. neumanni 1	Freilich et al. (2014)	KF380313	NA	KF379938	KF379848	KF380653	KF380128	N8° 51.3351' E38° 48.1215'	Ethiopia
XF-216	P. neumanni 1	Freilich et al. (2014)	KF380314	NA	KF379939	NA	NA	KF380129	N8° 51.3351' E38° 48.1215'	Ethiopia
XF-401	P. neumanni 1	Freilich et al. (2014)	NA	NA	KF379987	KF379849	KF380654	KF380165	N7° 50.1874' E37°21.3103'	Ethiopia
XF-410	P. neumanni 1	Freilich et al. (2014)	NA	NA	KF379988	KF379850	NA	NA	N8° 23.3087' E37°51.8200'	Ethiopia
XF-411	P. neumanni 1	Freilich et al. (2014)	NA	NA	NA	KF379851	KF380655	NA	N8° 23.3087' E37°51.8200'	Ethiopia
XF-412	P. neumanni 1	Freilich et al. (2014)	NA	NA	KF379989	KF379852	KF380656	KF380166	N8° 23.3087' E37°51.8200'	Ethiopia
XF-413	P. neumanni 1	Freilich et al. (2014)	NA	NA	KF379990	KF379853	NA	KF380167	N8° 23.3087' E37°51.8200'	Ethiopia
XF-415	P. neumanni 1	Freilich et al. (2014)	NA	NA	KF379991	KF379854	NA	NA	N8° 23.3087' E37°51.8200'	Ethiopia
XF-416	P. neumanni 1	Freilich et al. (2014)	KF380350	NA	KF379992	KF379855	KF380657	KF380168	N8° 23.3087' E37°51.8200'	Ethiopia
XF-417	P. neumanni 1	Freilich et al. (2014)	KF380351	NA	KF379993	KF379856	KF380658	KF380169	N8° 23.3087' E37°51.8200'	Ethiopia
XF-418	P. neumanni 1	Freilich et al. (2014)	KF380352	NA	KF379994	KF379857	NA	KF380170	N8° 50.8732' E38°24.4037'	Ethiopia
XF-419	P. neumanni 1	Freilich et al. (2014)	KF380353	NA	KF379995	KF379858	KF380659	KF380171	N8° 50.8732' E38°24.4037'	Ethiopia
XF-423	P. neumanni 1	Freilich et al. (2014)	KF380354	NA	KF379996	KF379859	KF380660	KF380172	N7° 51.8722' E37°22.3472'	Ethiopia
XF-424	P. neumanni 1	Freilich et al. (2014)	KF380355	NA	KF379997	KF379860	KF380661	KF380173	N7° 51.8722' E37°22.3472'	Ethiopia
XF-433	P. neumanni 1	Freilich et al. (2014)	KF380356	NA	KF379998	KF379861	NA	KF380174	N7° 38.9603' E36°50.6993'	Ethiopia
XF-434	P. neumanni 1	Freilich et al. (2014)	KF380357	NA	NA	KF379862	KF380662	KF380175	N7° 38.9603' E36°50.6993'	Ethiopia
XF-435	P. neumanni 1	Freilich et al. (2014)	KF380358	NA	KF379999	KF379863	NA	KF380176	N7° 38.9603' E36°50.6993'	Ethiopia
XF-436	P. neumanni 1	Freilich et al. (2014)	KF380359	NA	KF380000	KF379864	KF380663	KF380177	N7° 38.9603' E36°50.6993'	Ethiopia
XF-437	P. neumanni 1	Freilich et al. (2014)	KF380360	NA	NA	KF379865	KF380664	KF380178	N7° 38.9603' E36°50.6993'	Ethiopia
XF-438	P. neumanni 1	Freilich et al. (2014)	KF380361	NA	KF380001	KF379866	KF380665	KF380179	N7° 38.9603' E36°50.6993'	Ethiopia
XF-439	P. neumanni 1	Freilich et al. (2014)	KF380362	NA	KF380002	KF379867	KF380666	KF380180	N7° 38.9603' E36°50.6993'	Ethiopia
XF-450	P. neumanni 1	Freilich et al. (2014)	KF380364	NA	KF380004	KF379868	KF380667	NA	N7° 33.9997' E36°39.4504'	Ethiopia

XF-451	P. neumanni 1	Freilich et al. (2014)	NA	NA	KF380005	KF379869	KF380668	KF380182	N7° 33.9997' E36°39.4504'	Ethiopia
XF-473	P. neumanni 1	Freilich et al. (2014)	KF380366	NA	NA	NA	NA	NA	N7° 35.0246' E36°42.3433'	Ethiopia
XF-474	P. neumanni 1	Freilich et al. (2014)	KF380367	NA	NA	KF379870	KF380669	KF380185	N7° 35.0246' E36°42.3433'	Ethiopia
XF-475	P. neumanni 1	Freilich et al. (2014)	KF380368	NA	KF380009	KF379871	KF380670	KF380186	N7° 35.0246' E36°42.3433'	Ethiopia
XF-476	P. neumanni 1	Freilich et al. (2014)	KF380369	NA	KF380010	KF379872	KF380671	KF380187	N7° 35.0246' E36°42.3433'	Ethiopia
XF-489	P. neumanni 1	Freilich et al. (2014)	KF380371	NA	KF380011	KF379873	NA	KF380188	N7° 35.0246' E36°42.3433'	Ethiopia
XF-490	P. neumanni 1	Freilich et al. (2014)	KF380372	NA	KF380012	KF379874	KF380672	KF380189	N7° 35.0246' E36°42.3433'	Ethiopia
XF-491	P. neumanni 1	Freilich et al. (2014)	KF380373	NA	KF380013	KF379875	KF380673	KF380190	N7° 35.0246' E36°42.3433'	Ethiopia
XF-492	P. neumanni 1	Freilich et al. (2014)	KF380374	NA	KF380014	KF379876	NA	KF380191	N7° 35.0246' E36°42.3433'	Ethiopia
XF-562	P. neumanni 1	Freilich et al. (2014)	KF380378	NA	KF380018	KF379877	KF380674	KF380195	N8° 56.2327' E36°44.1855'	Ethiopia
XF-563	P. neumanni 1	Freilich et al. (2014)	KF380379	NA	KF380019	KF379878	KF380675	KF380196	N8° 56.2327' E36°44.1855'	Ethiopia
XF-565	P. neumanni 1	Freilich et al. (2014)	NA	NA	KF380020	KF379879	KF380676	KF380197	N8° 56.2327' E36°44.1855'	Ethiopia
XF-569	P. neumanni 1	Freilich et al. (2014)	KF380380	NA	KF380021	KF379880	KF380677	KF380198	N8° 56.2327' E36°44.1855'	Ethiopia
XF-570	P. neumanni 1	Freilich et al. (2014)	KF380381	NA	KF380022	NA	KF380678	NA	N8° 56.2327' E36°44.1855'	Ethiopia
XF-571	P. neumanni 1	Freilich et al. (2014)	KF380382	NA	KF380023	KF379881	KF380679	KF380199	N8° 56.2327' E36°44.1855'	Ethiopia
XF-573	P. neumanni 1	Freilich et al. (2014)	KF380383	NA	KF380024	KF379882	KF380680	KF380200	N8° 56.2327' E36°44.1855'	Ethiopia
XF-574	P. neumanni 1	Freilich et al. (2014)	KF380384	NA	KF380025	KF379883	KF380681	KF380201	N8° 56.2327' E36°44.1855'	Ethiopia
XF-589	P. neumanni 1	Freilich et al. (2014)	KF380390	NA	KF380034	KF379884	NA	KF380210	N7° 43.4493' E36°48.1724'	Ethiopia
XF-590	P. neumanni 1	Freilich et al. (2014)	KF380391	NA	KF380035	KF379885	NA	KF380211	N7° 43.4493' E36°48.1724'	Ethiopia
XF-591	P. neumanni 1	Freilich et al. (2014)	KF380392	NA	KF380036	KF379886	NA	KF380212	N7° 43.4493' E36°48.1724'	Ethiopia
XF-592	P. neumanni 1	Freilich et al. (2014)	KF380393	NA	KF380037	KF379887	NA	KF380213	N7° 43.4493' E36°48.1724'	Ethiopia
XF-593	P. neumanni 1	Freilich et al. (2014)	KF380394	NA	KF380038	NA	KF380682	KF380214	N7° 43.4493' E36°48.1724'	Ethiopia
XF-622	P. neumanni 1	Freilich et al. (2014)	KF380406	NA	KF380044	KF379888	KF380683	KF380224	N7° 32.6714' E36°34.6606'	Ethiopia
XF-127	P. neumanni 2	Freilich et al. (2014)	KF380292	NA	KF379913	KF379788	KF380618	KF380107	N9° 14.9732' E38° 45.6741	Ethiopia
XF-240	P. neumanni 2	Freilich et al. (2014)	KF380317	NA	NA	NA	NA	KF380131	N9° 38.2313' E39° 30.2933'	Ethiopia
XF-241	P. neumanni 2	Freilich et al. (2014)	KF380318	NA	KF379948	KF379789	NA	KF380132	N9° 38.2313' E39° 30.2933'	Ethiopia
XF-242	P. neumanni 2	Freilich et al. (2014)	KF380319	NA	KF379949	NA	NA	NA	N9° 38.2313' E39° 30.2933'	Ethiopia
XF-243	P. neumanni 2	Freilich et al. (2014)	NA	NA	KF379950	KF379790	KF380619	NA	N9° 38.2313' E39° 30.2933'	Ethiopia
XF-244	P. neumanni 2	Freilich et al. (2014)	KF380320	NA	KF379951	KF379791	KF380620	KF380133	N9° 38.2313' E39° 30.2933'	Ethiopia
XF-245	P. neumanni 2	Freilich et al. (2014)	KF380321	NA	KF379952	KF379792	NA	KF380134	N9° 38.2313' E39° 30.2933'	Ethiopia
XF-246	P. neumanni 2	Freilich et al. (2014)	NA	NA	NA	KF379793	KF380621	NA	N9° 38.2313' E39° 30.2933'	Ethiopia
XF-247	P. neumanni 2	Freilich et al. (2014)	KF380322	NA	KF379953	KF379794	NA	KF380135	N9° 38.2313' E39° 30.2933'	Ethiopia
XF-248	P. neumanni 2	Freilich et al. (2014)	KF380323	NA	KF379954	KF379795	NA	KF380136	N9° 38.2313' E39° 30.2933'	Ethiopia

XF-261	P. neumanni 2	Freilich et al. (2014)	KF380324	NA	KF379955	KF379796	KF380622	KF380137	N9° 35.0046' E39° 44.3387'	Ethiopia
XF-262	P. neumanni 2	Freilich et al. (2014)	NA	NA	NA	NA	NA	NA	N9° 35.0046' E39° 44.3387'	Ethiopia
XF-263	P. neumanni 2	Freilich et al. (2014)	KF380325	NA	KF379956	KF379797	KF380623	KF380138	N9° 35.0046' E39° 44.3387'	Ethiopia
XF-264	P. neumanni 2	Freilich et al. (2014)	KF380326	NA	KF379957	KF379798	KF380624	KF380139	N9° 35.0046' E39° 44.3387'	Ethiopia
XF-265	P. neumanni 2	Freilich et al. (2014)	NA	NA	NA	KF379799	KF380625	NA	N9° 35.0046' E39° 44.3387'	Ethiopia
XF-266	P. neumanni 2	Freilich et al. (2014)	KF380327	NA	KF379958	KF379800	KF380626	KF380140	N9° 35.0046' E39° 44.3387'	Ethiopia
XF-293	P. neumanni 2	Freilich et al. (2014)	KF380329	NA	KF379960	KF379801	NA	KF380142	N9° 35.0046' E39° 44.3387'	Ethiopia
XF-294	P. neumanni 2	Freilich et al. (2014)	NA	NA	KF379961	KF379802	NA	KF380143	N9° 35.0046' E39° 44.3387'	Ethiopia
XF-295	P. neumanni 2	Freilich et al. (2014)	NA	NA	KF379962	KF379803	KF380627	KF380144	N9° 35.0046' E39° 44.3387'	Ethiopia
XF-296	P. neumanni 2	Freilich et al. (2014)	KF380330	NA	KF379963	KF379804	NA	KF380145	N9° 35.0046' E39° 44.3387'	Ethiopia
XF-297	P. neumanni 2	Freilich et al. (2014)	KF380331	NA	KF379964	KF379805	NA	KF380146	N9° 35.0046' E39° 44.3387'	Ethiopia
XF-327	P. neumanni 2	Freilich et al. (2014)	KF380338	NA	KF379973	KF379806	KF380628	KF380151	N9° 509398' E39°45.4200'	Ethiopia
XF-368	P. neumanni 2	Freilich et al. (2014)	NA	NA	KF379974	KF379807	NA	KF380152	N10°24.0263' E39°39.1107'	Ethiopia
XF-369	P. neumanni 2	Freilich et al. (2014)	KF380339	NA	KF379975	KF379808	NA	KF380153	N10°24.0263' E39°39.1107'	Ethiopia
XF-370	P. neumanni 2	Freilich et al. (2014)	KF380340	NA	KF379976	KF379809	KF380629	KF380154	N10°24.0263' E39°39.1107'	Ethiopia
XF-371	P. neumanni 2	Freilich et al. (2014)	KF380341	NA	KF379977	KF379810	NA	KF380155	N10°24.0263' E39°39.1107'	Ethiopia
XF-372	P. neumanni 2	Freilich et al. (2014)	NA	NA	KF379978	KF379811	NA	KF380156	N10°24.0263' E39°39.1107'	Ethiopia
XF-373	P. neumanni 2	Freilich et al. (2014)	KF380342	NA	KF379979	KF379812	KF380630	KF380157	N10°24.0263' E39°39.1107'	Ethiopia
XF-374	P. neumanni 2	Freilich et al. (2014)	KF380343	NA	KF379980	KF379813	KF380631	KF380158	N10°24.0263' E39°39.1107'	Ethiopia
XF-375	P. neumanni 2	Freilich et al. (2014)	KF380344	NA	KF379981	KF379814	NA	KF380159	N10°24.0263' E39°39.1107'	Ethiopia
XF-377	P. neumanni 2	Freilich et al. (2014)	KF380345	NA	KF379982	KF379815	NA	KF380160	N10°24.0263' E39°39.1107'	Ethiopia
XF-378	P. neumanni 2	Freilich et al. (2014)	KF380346	NA	KF379983	KF379816	KF380632	KF380161	N10°24.0263' E39°39.1107'	Ethiopia
XF-379	P. neumanni 2	Freilich et al. (2014)	KF380347	NA	KF379984	KF379817	NA	KF380162	N10°24.0263' E39°39.1107'	Ethiopia
XF-380	P. neumanni 2	Freilich et al. (2014)	KF380348	NA	KF379985	KF379818	KF380633	KF380163	N10°24.0263' E39°39.1107'	Ethiopia
XF-821	P. neumanni 2	Freilich et al. (2014)	KF380415	NA	KF380051	KF379819	NA	KF380234	N7°3.0150' E38°49.0367'	Ethiopia
XF-822	P. neumanni 2	Freilich et al. (2014)	NA	NA	KF380052	NA	NA	NA	N7°3.0150' E38°49.0367'	Ethiopia
XF-823	P. neumanni 2	Freilich et al. (2014)	KF380416	NA	KF380053	KF379820	KF380634	KF380235	N7°3.0150' E38°49.0367'	Ethiopia
XF-824	P. neumanni 2	Freilich et al. (2014)	KF380417	NA	NA	NA	NA	KF380236	N7°3.0150' E38°49.0367'	Ethiopia
XF-825	P. neumanni 2	Freilich et al. (2014)	KF380418	NA	KF380054	NA	NA	NA	N7°3.0150' E38°49.0367'	Ethiopia
XF-826	P. neumanni 2	Freilich et al. (2014)	KF380419	NA	KF380055	NA	NA	KF380237	N7°3.0150' E38°49.0367'	Ethiopia
XF-827	P. neumanni 2	Freilich et al. (2014)	KF380420	NA	KF380056	NA	NA	KF380238	N7°3.0150' E38°49.0367'	Ethiopia
XF-832	P. neumanni 2	Freilich et al. (2014)	KF380421	NA	KF380057	NA	KF380635	KF380239	N7°3.0150' E38°49.0367'	Ethiopia
XF-839	P. neumanni 2	Freilich et al. (2014)	KF380422	NA	KF380058	NA	KF380636	KF380240	N7°3.0150' E38°49.0367'	Ethiopia

XF-84	P. neumanni 2	Freilich et al. (2014)	KF380423	NA	KF380062	KF379821	KF380637	KF380241	N9°1.9635' E38°39.9889'	Ethiopia
XF-840	P. neumanni 2	Freilich et al. (2014)	KF380424	NA	KF380059	KF379822	KF380638	KF380242	N7°3.0150' E38°49.0367'	Ethiopia
XF-841	P. neumanni 2	Freilich et al. (2014)	KF380425	NA	KF380060	KF379823	KF380639	KF380243	N7°3.0150' E38°49.0367'	Ethiopia
XF-845	P. neumanni 2	Freilich et al. (2014)	KF380426	NA	KF380061	NA	KF380640	KF380244	N7°3.0150' E38°49.0367'	Ethiopia
XF-85	P. neumanni 2	Freilich et al. (2014)	KF380427	NA	KF380063	KF379824	NA	KF380245	N9°1.9635'E38°39.9889'	Ethiopia
XF-86	P. neumanni 2	Freilich et al. (2014)	KF380428	NA	KF380068	KF379825	NA	KF380246	N9°1.9635'E38°39.9889'	Ethiopia
XF-863	P. neumanni 2	Freilich et al. (2014)	KF380429	NA	NA	KF379826	KF380641	KF380247	N7°51.4045' E38°53.3662'	Ethiopia
XF-864	P. neumanni 2	Freilich et al. (2014)	KF380430	NA	KF380064	KF379827	KF380642	KF380248	N7°51.4045' E38°53.3662'	Ethiopia
XF-865	P. neumanni 2	Freilich et al. (2014)	KF380431	NA	KF380065	KF379828	KF380643	KF380249	N7°51.4045' E38°53.3662'	Ethiopia
XF-866	P. neumanni 2	Freilich et al. (2014)	KF380432	NA	KF380066	NA	KF380644	KF380250	N7°51.4045' E38°53.3662'	Ethiopia
XF-867	P. neumanni 2	Freilich et al. (2014)	KF380433	NA	KF380067	KF379829	KF380645	KF380251	N7°51.4045' E38°53.3662'	Ethiopia
XF-87	P. neumanni 2	Freilich et al. (2014)	KF380434	NA	KF380073	KF379830	NA	KF380252	N9°1.9635' E38°39.9889'	Ethiopia
XF-877	P. neumanni 2	Freilich et al. (2014)	KF380435	NA	KF380070	KF379831	NA	KF380253	N7°51.7521' E38°57.0436'	Ethiopia
XF-878	P. neumanni 2	Freilich et al. (2014)	KF380436	NA	KF380071	KF379832	NA	KF380254	N7°51.7521' E38°57.0436'	Ethiopia
XF-879	P. neumanni 2	Freilich et al. (2014)	KF380437	NA	KF380072	KF379833	KF380646	KF380255	N7°51.7521' E38°57.0436'	Ethiopia
XF-880	P. neumanni 2	Freilich et al. (2014)	KF380438	NA	KF380074	KF379834	KF380647	KF380256	N7°51.7521' E38°57.0436'	Ethiopia
XF-881	P. neumanni 2	Freilich et al. (2014)	KF380439	NA	KF380075	NA	NA	KF380257	N7°51.7521' E38°57.0436'	Ethiopia
XF-882	P. neumanni 2	Freilich et al. (2014)	KF380440	NA	KF380076	KF379835	KF380648	KF380280	N7°51.7521' E38°57.0436'	Ethiopia
XF-948	P. neumanni 2	Freilich et al. (2014)	KF380458	NA	KF380098	KF379836	NA	KF380281	N7°55.0820' E39°7.4528'	Ethiopia
XF-949	P. neumanni 2	Freilich et al. (2014)	KF380459	NA	KF380099	KF379837	KF380649	KF380282	N7°51.0249' E39°8.1737'	Ethiopia
XF-950	P. neumanni 2	Freilich et al. (2014)	KF380460	NA	KF380100	KF379838	NA	KF380283	N7°51.0249' E39°8.1737'	Ethiopia
XF-951	P. neumanni 2	Freilich et al. (2014)	KF380461	NA	KF380101	NA	NA	KF380284	N7°51.0249' E39°8.1737'	Ethiopia
XF-952	P. neumanni 2	Freilich et al. (2014)	KF380462	NA	NA	NA	NA	KF380285	N7°51.0249' E39°8.1737'	Ethiopia
XF-953	P. neumanni 2	Freilich et al. (2014)	KF380463	NA	KF380102	KF379839	KF380650	KF380286	N7°51.0249' E39°8.1737'	Ethiopia
XF-954	P. neumanni 2	Freilich et al. (2014)	KF380464	NA	KF380103	NA	NA	KF380268	N7°51.0249' E39°8.1737'	Ethiopia
XF-923	P. neumanni 3	Freilich et al. (2014)	KF380445	NA	KF380085	KF379889	KF380684	KF380272	N7°6.6736' E39°44.5788'	Ethiopia
XF-927	P. neumanni 3	Freilich et al. (2014)	KF380449	NA	KF380089	KF379890	KF380685	KF380181	N7°6.6736' E39°44.5788'	Ethiopia
XF-44-10	P. neumanni 4	Freilich et al. (2014)	KF380363	NA	KF380003	KF379900	KF380697	KF380227	N6°42.5852' E39°43.3677'	Ethiopia
XF-781	P. neumanni 4	Freilich et al. (2014)	KF380409	NA	KF380045	KF379901	KF380698	KF380228	N5°48.1639' E38°16.2302'	Ethiopia
XF-782	P. neumanni 4	Freilich et al. (2014)	KF380410	NA	NA	KF379902	KF380699	KF380229	N5°48.1639' E38°16.2302'	Ethiopia
XF-783	P. neumanni 4	Freilich et al. (2014)	NA	NA	KF380046	KF379903	KF380700	KF380230	N5°48.1639' E38°16.2302'	Ethiopia
XF-816	P. neumanni 4	Freilich et al. (2014)	KF380411	NA	KF380047	KF379904	KF380701	KF380231	N5°48.1639' E38°16.2302'	Ethiopia
XF-817	P. neumanni 4	Freilich et al. (2014)	KF380412	NA	KF380048	KF379905	KF380702	KF380232	N5°48.1639' E38°16.2302'	Ethiopia

XF-818	P. neumanni 4	Freilich et al. (2014)	KF380413	NA	KF380049	KF379906	KF380704	KF380233	N5°48.1639' E38°16.2302'	Ethiopia
XF-819	P. neumanni 5	Freilich et al. (2014)	KF380414	NA	KF380050	KF379907	KF380705	KF380111	N5°48.1639' E38°16.2302'	Ethiopia
XF-140	P. neumanni 5	Freilich et al. (2014)	KF380296	NA	KF379918	KF379891	KF380686	KF380112	N10° 11.4467' E38° 8.4044'	Ethiopia
XF-141	P. neumanni 5	Freilich et al. (2014)	NA	NA	KF379919	NA	KF380706	NA	N10° 11.4467' E38° 8.4044'	Ethiopia
XF-142	P. neumanni 5	Freilich et al. (2014)	NA	NA	KF379920	NA	NA	NA	N10° 11.4467' E38° 8.4044'	Ethiopia
XF-143	P. neumanni 5	Freilich et al. (2014)	NA	NA	NA	NA	NA	KF380113	N10° 11.4467' E38° 8.4044'	Ethiopia
XF-146	P. neumanni 5	Freilich et al. (2014)	KF380297	NA	KF379921	NA	KF380687	NA	N10° 11.4467' E38° 8.4044'	Ethiopia
XF-147	P. neumanni 5	Freilich et al. (2014)	KF380298	NA	NA	NA	NA	KF380114	N10° 11.4467' E38° 8.4044'	Ethiopia
XF-148	P. neumanni 5	Freilich et al. (2014)	KF380299	NA	KF379922	NA	KF380703	KF380115	N10° 11.4467' E38° 8.4044'	Ethiopia
XF-149	P. neumanni 5	Freilich et al. (2014)	KF380300	NA	KF379923	KF379892	KF380688	KF380116	N10° 11.4467' E38° 8.4044'	Ethiopia
XF-150	P. neumanni 5	Freilich et al. (2014)	NA	NA	KF379924	KF379893	KF380689	NA	N10° 14.4060' E37° 58.1187'	Ethiopia
XF-151	P. neumanni 5	Freilich et al. (2014)	NA	NA	KF379925	KF379894	NA	KF380117	N10° 14.4060' E37° 58.1187'	Ethiopia
XF-152	P. neumanni 5	Freilich et al. (2014)	KF380301	NA	KF379926	NA	NA	KF380118	N10° 14.4060' E37° 58.1187'	Ethiopia
XF-153	P. neumanni 5	Freilich et al. (2014)	NA	NA	KF379927	KF379895	NA	KF380119	N10° 14.4060' E37° 58.1187'	Ethiopia
XF-154	P. neumanni 5	Freilich et al. (2014)	KF380302	NA	KF379928	NA	KF380690	KF380120	N10° 13.3793' E37° 59.2569'	Ethiopia
XF-155	P. neumanni 5	Freilich et al. (2014)	KF380303	NA	KF379929	KF379896	KF380691	KF380121	N10° 13.3793' E37° 59.2569'	Ethiopia
XF-169	P. neumanni 5	Freilich et al. (2014)	KF380304	NA	KF379930	KF379897	KF380692	NA	N10° 21.2362' E37° 42.7307'	Ethiopia
XF-171	P. neumanni 5	Freilich et al. (2014)	KF380305	NA	KF379931	NA	KF380693	KF380122	N10° 21.2362' E37° 42.7307'	Ethiopia
XF-172	P. neumanni 5	Freilich et al. (2014)	KF380306	NA	KF379932	KF379898	KF380694	KF380123	N10° 21.2362' E37° 42.7307'	Ethiopia
XF-173	P. neumanni 5	Freilich et al. (2014)	KF380307	NA	KF379933	KF379899	KF380695	NA	N10° 21.2362' E37° 42.7307'	Ethiopia
XF-174	P. cooperi	Freilich et al. (2014)	KF380308	NA	KF379934	NA	KF380696	KF380105	N10° 21.2362' E37° 42.7307'	Ethiopia
XF-100	P. cooperi	Freilich et al. (2014)	KF380289	NA	KF379908	KF379766	KF380579	NA	N9°29.8647' E38°52.4750'	Ethiopia
XF-103	P. cooperi	Freilich et al. (2014)	NA	NA	KF379909	NA	KF380580	NA	N9°29.8647' E38°52.4750'	Ethiopia
XF-106	P. neumanni 4	Freilich et al. (2014)	NA	NA	KF379910	NA	KF380581	NA	N9°29.8647' E38°52.4750'	Ethiopia
XF-111	P. cooperi	Freilich et al. (2014)	NA	NA	KF379911	NA	KF380582	KF380106	N9°29.8647' E38°52.4750'	Ethiopia
XF-112	P. cooperi	Freilich et al. (2014)	KF380290	NA	KF379912	NA	KF380583	NA	N9°29.8647' E38°52.4750'	Ethiopia
XF-113	P. cooperi	Freilich et al. (2014)	NA	NA	NA	NA	KF380584	NA	N9°29.8647' E38°52.4750'	Ethiopia
XF-123	P. cooperi	Freilich et al. (2014)	KF380291	NA	KF379940	NA	NA	NA	N9°29.8647' E38°52.4750'	Ethiopia
XF-222	P. cooperi	Freilich et al. (2014)	KF380315	NA	KF379941	NA	KF380585	NA	N9° 5.8765' E38° 57.5962'	Ethiopia
XF-223	P. cooperi	Freilich et al. (2014)	NA	NA	KF379942	NA	NA	NA	N9° 5.8765' E38° 57.5962'	Ethiopia
XF-224	P. cooperi	Freilich et al. (2014)	NA	NA	KF379943	NA	NA	NA	N9° 5.8765' E38° 57.5962'	Ethiopia

XF-225	P. cooperi	Freilich et al. (2014)	NA	NA	KF379944	KF379767	KF380586	NA	N9° 7.6190' E38° 59.8490'	Ethiopia
XF-231	P. cooperi	Freilich et al. (2014)	NA	NA	KF379945	NA	NA	KF380130	N9° 28.5252' E39° 24.2726'	Ethiopia
XF-232	P. cooperi	Freilich et al. (2014)	NA	NA	KF379946	NA	KF380587	NA	N9° 28.5252' E39° 24.2726'	Ethiopia
XF-233	P. cooperi	Freilich et al. (2014)	KF380316	NA	KF379947	NA	NA	NA	N9° 28.5252' E39° 24.2726'	Ethiopia
XF-237	P. cooperi	Freilich et al. (2014)		NA	KF379959	NA	NA	KF380141	N9° 33.8990' E39° 28.8398'	Ethiopia
XF-269	P. cooperi	Freilich et al. (2014)	KF380328	NA	NA	NA	KF380588	NA	N9° 39.0094' E39° 37.5138'	Ethiopia
XF-298	P. cooperi	Freilich et al. (2014)	KF380332	NA	KF379965	NA	NA	KF380147	N9° 40.5198' E39° 34.5870'	Ethiopia
XF-299	P. cooperi	Freilich et al. (2014)	KF380333	NA	KF379966	NA	KF380589	KF380148	N9° 40.5198' E39° 34.5870'	Ethiopia
XF-300	P. cooperi	Freilich et al. (2014)	KF380334	NA	NA	KF379768	NA	NA	N9° 40.5198' E39° 34.5870'	Ethiopia
XF-301	P. cooperi	Freilich et al. (2014)	NA	NA	NA	NA	NA	NA	N9° 39.0261' E39° 37.4229'	Ethiopia
XF-303	P. cooperi	Freilich et al. (2014)	KF380335	NA	KF379967	NA	KF380590	NA	N9° 39.0261' E39° 37.4229'	Ethiopia
XF-304	P. cooperi	Freilich et al. (2014)	KF380336	NA	KF379968	NA	NA	NA	N9° 39.0261' E39° 37.4229'	Ethiopia
XF-305	P. cooperi	Freilich et al. (2014)	NA	NA	KF379969	NA	KF380591	NA	N9° 39.0261' E39° 37.4229'	Ethiopia
XF-306	P. cooperi	Freilich et al. (2014)	NA	NA	KF379970	NA	KF380592	KF380149	N9° 39.0261' E39° 37.4229'	Ethiopia
XF-307	P. cooperi	Freilich et al. (2014)	NA	NA	KF379971	NA	NA	NA	N9° 39.0261' E39° 37.4229'	Ethiopia
XF-310	P. cooperi	Freilich et al. (2014)	KF380337	NA	KF379972	NA	NA	KF380150	N9° 39.0261' E39° 37.4229'	Ethiopia
XF-313	P. cooperi	Freilich et al. (2014)	KF380349	NA	KF379986	NA	KF380593	KF380164	N9° 39.0261' E39° 37.4229'	Ethiopia
XF-381	P. cooperi	Freilich et al. (2014)	NA	NA	KF380069	KF379769	KF380594	NA	N10°24.0263' E39°39.1107'	Ethiopia
XF-872	P. cooperi	Freilich et al. (2014)	NA	NA	NA	NA	NA	KF380258	N7°51.7521' E38°57.0436'	Ethiopia
XF-884	P. cooperi	Freilich et al. (2014)	NA	NA	KF380077	NA	NA	KF380259	N6°59.7942' E39°17.7658'	Ethiopia
XF-885	P. cooperi	Freilich et al. (2014)	NA	NA	KF380078	NA	NA	KF380260	N6°59.7942' E39°17.7658'	Ethiopia
XF-886	P. cooperi	Freilich et al. (2014)	KF380441	NA	KF380079	NA	NA	KF380261	N6°59.7942' E39°17.7658'	Ethiopia
XF-892	P. cooperi	Freilich et al. (2014)	NA	NA	KF380081	KF379770	NA	KF380263	N6°59.7942' E39°17.7658'	Ethiopia
XF-918	P. cooperi	Freilich et al. (2014)	NA	NA	KF380082	KF379771	KF380595	KF380264	N7°6.6736' E39°44.5788'	Ethiopia
XF-919	P. cooperi	Freilich et al. (2014)	KF380443	NA	KF380083	NA	NA	KF380265	N7°6.6736' E39°44.5788'	Ethiopia
XF-920	P. cooperi	Freilich et al. (2014)	NA	NA	KF380084	NA	NA	KF380266	N7°6.6736' E39°44.5788'	Ethiopia
XF-921	P. cooperi	Freilich et al. (2014)	KF380456	NA	KF380096	NA	NA	KF380279	N7°6.6736' E39°44.5788'	Ethiopia
XF-935	P. cooperi	Freilich et al. (2014)	KF380457	NA	NA	NA	NA	NA	N7°21.6832' E39°15.5177'	Ethiopia
XF-936	P. cooperi	Freilich et al. (2014)	NA	NA	KF380097	NA	NA	NA	N7°21.6832' E39°15.5177'	Ethiopia
XF-937	P. cooperi	Freilich et al. (2014)	NA	NA	NA	KF379772	NA	KF380287	N7°21.6832' E39°15.5177'	Ethiopia
XF-97	P. cooperi	Freilich et al. (2014)	NA	NA	NA	KF379773	KF380596	KF380288	N9°29.8647' E38°52.4750'	Ethiopia
XF-98	P. cooperi	Freilich et al. (2014)	NA	NA	KF380104	NA	NA	NA	N9°29.8647' E38°52.4750'	Ethiopia

XF-99	P. cooperi	Freilich et al. (2014)	KF380465	NA	NA	NA	KF380597	KF380183	N9°29.8647' E38°52.4750'	Ethiopia
XF-460	P. erlangeri	Freilich et al. (2014)	NA	NA	KF380006	KF379774	KF380598	NA	N7° 32.3979' E36°34.3775'	Ethiopia
XF-461	P. erlangeri	Freilich et al. (2014)	NA	NA	KF380007	NA	NA	NA	N7° 32.3979' E36°34.3775'	Ethiopia
XF-462	P. erlangeri	Freilich et al. (2014)	KF380365	NA	KF380008	KF379775	KF380599	KF380184	N7° 32.3979' E36°34.3775'	Ethiopia
XF-478	P. erlangeri	Freilich et al. (2014)	KF380370	NA	NA	NA	NA	NA	N7° 35.0246' E36°42.3433'	Ethiopia
XF-493	P. erlangeri	Freilich et al. (2014)	KF380375	NA	KF380015	NA	KF380600	KF380192	N7° 32.3974' E36°34.3780'	Ethiopia
XF-494	P. erlangeri	Freilich et al. (2014)	KF380376	NA	KF380016	KF379776	KF380601	KF380193	N7° 32.3974' E36°34.3780'	Ethiopia
XF-495	P. erlangeri	Freilich et al. (2014)	KF380377	NA	KF380017	KF379777	KF380602	KF380194	N7° 32.3974' E36°34.3780'	Ethiopia
XF-578	P. erlangeri	Freilich et al. (2014)	KF380385	NA	NA	NA	KF380603	KF380202	N7° 17.0010' E36°13.8647'	Ethiopia
XF-579	P. erlangeri	Freilich et al. (2014)	KF380386	NA	NA	KF379778	KF380604	NA	N7° 17.0010' E36°13.8647'	Ethiopia
XF-580	P. erlangeri	Freilich et al. (2014)	NA	NA	KF380026	KF379779	KF380605	NA	N7° 17.0010' E36°13.8647'	Ethiopia
XF-581	P. erlangeri	Freilich et al. (2014)	NA	NA	KF380027	KF379780	KF380606	KF380203	N7° 17.0010' E36°13.8647'	Ethiopia
XF-582	P. erlangeri	Freilich et al. (2014)	NA	NA	KF380028	NA	KF380607	KF380204	N7° 17.0010' E36°13.8647'	Ethiopia
XF-583	P. erlangeri	Freilich et al. (2014)	KF380387	NA	KF380029	KF379781	KF380608	KF380205	N7° 17.0010' E36°13.8647'	Ethiopia
XF-584	P. erlangeri	Freilich et al. (2014)	KF380388	NA	KF380030	NA	KF380609	KF380206	N7° 17.0010' E36°13.8647'	Ethiopia
XF-585	P. erlangeri	Freilich et al. (2014)	NA	NA	KF380031	KF379782	KF380610	KF380207	N7° 17.0010' E36°13.8647'	Ethiopia
XF-586	P. erlangeri	Freilich et al. (2014)	NA	NA	KF380032	NA	KF380611	KF380208	N7° 17.0010' E36°13.8647'	Ethiopia
XF-587	P. erlangeri	Freilich et al. (2014)	KF380389	NA	KF380033	NA	NA	KF380209	N7° 24.5726' E36°20.4232'	Ethiopia
XF-601	P. erlangeri	Freilich et al. (2014)	KF380395	NA	KF380039	NA	KF380612	KF380215	N6° 55.3867' E36°28.2329'	Ethiopia
XF-606	P. erlangeri	Freilich et al. (2014)	KF380396	NA	KF380040	KF379783	NA	KF380216	N6° 55.3867' E36°28.2329'	Ethiopia
XF-607	P. erlangeri	Freilich et al. (2014)	KF380397	NA	NA	NA	NA	NA	N6° 55.3867' E36°28.2329'	Ethiopia
XF-608	P. erlangeri	Freilich et al. (2014)	KF380398	NA	NA	NA	NA	NA	N6° 55.3867' E36°28.2329'	Ethiopia
XF-611	P. erlangeri	Freilich et al. (2014)	KF380399	NA	NA	NA	KF380613	KF380217	N7° 0.7613' E37°16.9372'	Ethiopia
XF-612	P. erlangeri	Freilich et al. (2014)	KF380400	NA	NA	NA	NA	NA	N7° 0.7613' E37°16.9372'	Ethiopia
XF-613	P. erlangeri	Freilich et al. (2014)	NA	NA	NA	NA	NA	KF380218	N7° 0.7613' E37°16.9372'	Ethiopia
XF-614	P. erlangeri	Freilich et al. (2014)	KF380401	NA	KF380041	KF379784	KF380614	KF380219	N7° 0.7613' E37°16.9372'	Ethiopia
XF-615	P. erlangeri	Freilich et al. (2014)	KF380402	NA	KF380042	KF379785	NA	KF380220	N7° 0.7613' E37°16.9372'	Ethiopia
XF-617	P. erlangeri	Freilich et al. (2014)	KF380403	NA	NA	NA	NA	KF380221	N7° 0.7613' E37°16.9372'	Ethiopia
XF-619	P. erlangeri	Freilich et al. (2014)	KF380404	NA	NA	NA	KF380615	KF380222	N7° 0.7613' E37°16.9372'	Ethiopia
XF-621	P. erlangeri	Freilich et al. (2014)	KF380405	NA	KF380043	KF379786	KF380616	KF380223	N7° 32.6714' E36°34.6606'	Ethiopia
XF-648	P. erlangeri	Freilich et al. (2014)	KF380407	NA	NA	KF379787	KF380617	KF380225	N6° 55.3867' E36°28.2329'	Ethiopia
XF-780	P. erlangeri	Freilich et al. (2014)	KF380408	NA	NA	NA	NA	KF380226	N5°48.1639' E38°16.2302'	Ethiopia
XF-917	P. nana	Freilich et al. (2014)	KF380442	NA	KF380080	KF379760	NA	KF380262	N7°6.6736' E39°44.5788'	Ethiopia

XF-922	P. nana	Freilich et al. (2014)	KF380444	NA	NA	NA	NA	KF380267	N7°6.6736' E39°44.5788'	Ethiopia
XF-925	P. nana	Freilich et al. (2014)	KF380447	NA	KF380087	NA	KF380573	KF380270	N7°6.6736' E39°44.5788'	Ethiopia
XF-926	P. nana	Freilich et al. (2014)	KF380448	NA	KF380088	KF379761	KF380574	KF380271	N7°6.6736' E39°44.5788'	Ethiopia
XF-929	P. nana	Freilich et al. (2014)	KF380450	NA	KF380090	NA	KF380575	KF380273	N7°8.4271' E39°54.9541'	Ethiopia
XF-930	P. nana	Freilich et al. (2014)	KF380451	NA	KF380091	KF379762	KF380576	KF380274	N7°8.4271' E39°54.9541'	Ethiopia
XF-931	P. nana	Freilich et al. (2014)	KF380452	NA	KF380092	KF379763	NA	KF380275	N7°8.4271' E39°54.9541'	Ethiopia
XF-932	P. nana	Freilich et al. (2014)	KF380453	NA	KF380093	KF379764	NA	KF380276	N7°8.4271' E39°54.9541'	Ethiopia
XF-933	P. nana	Freilich et al. (2014)	KF380454	NA	KF380094	NA	KF380577	KF380277	N7°8.4271' E39°54.9541'	Ethiopia
XF-934	P. nana	Freilich et al. (2014)	KF380455	NA	KF380095	KF379765	KF380578	KF380278	N7°8.4271' E39°54.9541'	Ethiopia
XF-924	P. nana / P. neumanni 3	Freilich et al. (2014)	KF380446	NA	KF380086	NA	KF380572	KF380269	N7°6.6736' E39°44.5788'	Ethiopia
TJC 212	P. anchietae	This study	MF078189	MF078284	NA	NA	NA	MF078103	N6°28.6775' E39°45.1973'	Ethiopia
TJC 218	P. neumanni 4	This study	MF078190	MF078285	MF078240	MF078147	MF078078	MF078104	N6°42.9870' E39°43.4903'	Ethiopia
TJC 219	P. neumanni 3	This study	MF078191	MF078286	MF078241	MF078148	MF078079	MF078105	N6°42.9870' E39°43.4903'	Ethiopia
TJC 220	P. neumanni 4	This study	MF078192	MF078287	MF078242	MF078149	MF078080	MF078106	N6°42.9870' E39°43.4903'	Ethiopia
TJC 224	P. neumanni 4	This study	MF078193	MF078288	MF078243	MF078150	MF078081	MF078107	N6°43.0673' E39°43.5431'	Ethiopia
TJC 240	P. neumanni 1	This study	MF078194	MF078289	MF078244	NA	MF078082	MF078108	N8°24.1601' E36°18.6233'	Ethiopia
TJC 241	P. neumanni 1	This study	MF078195	MF078290	MF078245	MF078151	MF078083	MF078109	N8°24.1601' E36°18.6233'	Ethiopia
TJC 244	P. neumanni 1	This study	MF078196	MF078291	MF078246	NA	MF078084	MF078110	N8°24.1601' E36°18.6233'	Ethiopia
TJC 245	P. neumanni 1	This study	MF078197	MF078292	MF078247	MF078152	NA	MF078111	N8°24.1601' E36°18.6233'	Ethiopia
TJC 247	P. neumanni 1	This study	MF078198	MF078293	MF078248	MF078153	NA	MF078112	N8°24.1523' E36°18.5760'	Ethiopia
TJC 254	P. erlangeri	This study	MF078199	MF078294	MF078249	MF078154	MF078085	MF078113	N8°24.1811' E36°18.6390'	Ethiopia
TJC 255	P. neumanni 1	This study	MF078200	MF078295	MF078250	MF078155	MF078086	MF078114	N8°24.1811' E36°18.6390'	Ethiopia
TJC 276	P. neumanni 1	This study	MF078201	MF078296	MF078251	MF078156	NA	MF078115	N8°23.9861' E36°18.5716'	Ethiopia
TJC 288	P. neumanni 1	This study	MF078202	MF078297	MF078252	MF078157	MF078087	MF078116	N8°24.1523' E36°18.5760'	Ethiopia
TJC 295	P. neumanni 1	This study	MF078203	MF078298	MF078253	MF078158	NA	MF078117	N8°24.1523' E36°18.5760'	Ethiopia
TJC 300	P. neumanni 1	This study	MF078204	MF078299	MF078254	MF078159	NA	NA	N8°24.1601' E36°18.6233'	Ethiopia
TJC 301	P. neumanni 1	This study	MF078205	MF078300	MF078255	MF078160	MF078088	NA	N8°24.1601' E36°18.6233'	Ethiopia
TJC 306	P. anchietae	This study	MF078206	MF078301	MF078256	MF078161	NA	MF078118	N4°59.0525' E36°51.4745'	Ethiopia
TJC 317	P. neumanni 1	This study	MF078207	MF078302	MF078257	NA	NA	MF078119	N7°46.4543' E36°43.6931'	Ethiopia
TJC 318	P. anchietae sp.	This study	MF078208	MF078303	NA	NA	NA	MF078120	N8°15.9341' E33°56.8126'	Ethiopia
TJC 319	P. anchietae sp.	This study	MF078209	MF078304	MF078258	MF078162	MF078089	MF078121	N8°15.9341' E33°56.8126'	Ethiopia
TJC 320	P. sp.	This study	MF078210	MF078305	MF078259	MF078163	NA	MF078122	N8°15.9341' E33°56.8126'	Ethiopia

TJC 321	P. anchietae sp.	This study	MF078211	MF078306	MF078260	MF078164	NA	MF078123	N8°15.9341' E33°56.8126'	Ethiopia
TJC 343	P. anchietae sp.	This study	MF078212	MF078307	MF078261	MF078165	MF078090	MF078124	N8°15.9341' E33°56.8126'	Ethiopia
TJC 344	P. anchietae sp.	This study	MF078213	NA	NA	NA	NA	NA	N8°15.9341' E33°56.8126'	Ethiopia
TJC 393	P. sp.	This study	MF078214	MF078308	NA	NA	NA	MF078125	N8°16.1250' E33°56.7695'	Ethiopia
TJC 410	P. sp.	This study	MF078215	MF078309	MF078262	MF078166	NA	MF078126	N8°16.0186' E33°56.6063'	Ethiopia
TJC 451	P. sp.	This study	MF078216	MF078310	MF078263	MF078167	NA	MF078127	N8°16.0871' E33°56.6880'	Ethiopia
TJC 455	P. sp.	This study	MF078217	MF078311	NA	NA	NA	MF078128	N8°16.1250' E33°56.7695'	Ethiopia
TJC 482	P. nilotica	This study	MF078218	NA	MF078264	NA	NA	MF078129	N7°5.4365' E38°28.7236'	Ethiopia
TJC 483	P. nilotica	This study	MF078219	MF078312	MF078265	MF078168	MF078091	MF078130	N7°5.4365' E38°28.7236'	Ethiopia
TJC 484	P. nilotica	This study	MF078220	NA	MF078266	MF078169	MF078092	MF078131	N7°5.4365' E38°28.7236'	Ethiopia
TJC 511	P. cooperi	This study	MF078221	MF078313	MF078267	MF078170	MF078093	MF078132	N7°7.3216' E39°43.7220'	Ethiopia
TJC 512	P. cooperi	This study	MF078222	MF078314	MF078268	MF078171	MF078094	MF078133	N7°7.3216' E39°43.7220'	Ethiopia
TJC 513	P. cooperi	This study	MF078223	MF078315	MF078269	MF078172	MF078095	MF078134	N7°7.3216' E39°43.7220'	Ethiopia
TJC 514	P. cooperi	This study	MF078224	MF078316	MF078270	MF078173	NA	MF078135	N7°7.3216' E39°43.7220'	Ethiopia
TJC 515	P. cooperi	This study	MF078225	MF078317	MF078271	MF078174	MF078096	MF078136	N7°7.2275' E39°43.1735'	Ethiopia
TJC 523	P. cooperi	This study	MF078226	MF078318	MF078272	MF078175	NA	MF078137	N7°5.7196' E40°3.8771'	Ethiopia
TJC 524	P. cooperi	This study	MF078227	MF078319	MF078273	MF078176	NA	MF078138	N7°5.7196' E40°3.8771'	Ethiopia
TJC 525	P. cooperi	This study	MF078228	MF078320	MF078274	MF078177	MF078097	MF078139	N7°5.7196' E40°3.8771'	Ethiopia
TJC 526	P. cooperi	This study	MF078229	MF078321	MF078275	MF078178	NA	MF078140	N7°5.7196' E40°3.8771'	Ethiopia
TJC 577	P. anchietae	This study	MF078230	MF078322	MF078276	MF078179	MF078098	MF078141	N5°30.2081' E39°42.0900'	Ethiopia
TJC 578	P. anchietae	This study	MF078231	MF078323	MF078277	MF078180	NA	MF078142	N5°30.2081' E39°42.0900'	Ethiopia
TJC 593	P. nilotica	This study	MF078232	NA	MF078278	MF078181	NA	MF078143	N6°35.1623' E38°23.3225'	Ethiopia
TJC 594	P. anchietae	This study	MF078233	MF078324	NA	MF078182	MF078099	NA	N6°35.1623' E38°23.3225'	Ethiopia
TJC 598	P. anchietae	This study	MF078234	MF078325	NA	MF078183	NA	MF078144	N5°30.2081' E39°42.0900'	Ethiopia
TJC 599	P. anchietae	This study	MF078235	MF078326	NA	MF078184	NA	NA	N5°30.2081' E39°42.0900'	Ethiopia
TJC 867	P. neumanni 4	This study	MF078236	MF078327	MF078279	NA	MF078100	MF078145	N6°39.8261' E39°43.7843'	Ethiopia
TJC 884	P. harenna	This study	NA	MF078328	MF078280	MF078185	NA	NA	N6°30.8098' E39°44.2805'	Ethiopia
TJC 886	P. harenna	This study	MF078237	MF078329	MF078281	MF078186	NA	NA	N6°41.5288' E39°40.1885'	Ethiopia
TJC 887	P. neumanni 4	This study	MF078238	MF078330	MF078282	MF078187	MF078101	NA	N6°41.5288' E39°40.1885'	Ethiopia
TJC 888	P. neumanni 4	This study	MF078239	MF078331	MF078283	MF078188	MF078102	MF078146	N6°41.5288' E39°40.1885'	Ethiopia
AF215342	Hemisus marmoratus	Vences (1999)	AF215342	NA	NA	NA	NA	NA	NA	NA
AF215403	P. oxyrhynchus	Vences (1999)	AF215403	NA	NA	NA	NA	NA	NA	NA

AF215404	P. anchietae	Vences (1999)	AF215404	NA	NA	NA	NA	NA	NA	NA
AF215405	P. anchietae	Vences (1999)	AF215405	NA	NA	NA	NA	NA	NA	NA
AF215406	P. mascareniensis	Vences (1999)	AF215406	NA	NA	NA	NA	NA	NA	NA
AF215407	P. mascareniensis	Vences (1999)	AF215407	NA	NA	NA	NA	NA	NA	NA
AF215408	P. bibroni	Vences (1999)	AF215408	NA	NA	NA	NA	NA	NA	NA
AF215409	P. oxyrhynchus	Vences (1999)	AF215409	NA	NA	NA	NA	NA	NA	NA
AF215410	P. subpunctata	Vences (1999)	AF215410	NA	NA	NA	NA	NA	NA	NA
AF215411	P. porosissima	Vences (1999)	AF215411	NA	NA	NA	NA	NA	NA	NA
AF261267	P. anchietae	Richards, C.M. (Unpublished)	AF261267	NA	NA	NA	NA	NA	NA	NA
AY517587	P. mascareniensis	Vences et al. (2004)	AY517587	NA	NA	NA	NA	NA	NA	Madagascar
AY517588	P. mascareniensis	Vences et al. (2004)	AY517588	NA	NA	NA	NA	NA	NA	Madagascar
AY517589	P. mascareniensis	Vences et al. (2004)	AY517589	NA	NA	NA	NA	NA	NA	Mauritius
AY517590	P. mascareniensis	Vences et al. (2004)	AY517590	NA	NA	NA	NA	NA	NA	Madagascar
AY517591	P. mascareniensis	Vences et al. (2004)	AY517591	NA	NA	NA	NA	NA	NA	Madagascar
AY517592	P. mascareniensis	Vences et al. (2004)	AY517592	NA	NA	NA	NA	NA	NA	Madagascar
AY517593	P. mascareniensis	Vences et al. (2004)	AY517593	NA	NA	NA	NA	NA	NA	Madagascar
AY517594	P. mascareniensis	Vences et al. (2004)	AY517594	NA	NA	NA	NA	NA	NA	Madagascar
AY517595	P. mascareniensis	Vences et al. (2004)	AY517595	NA	NA	NA	NA	NA	NA	Tanzania
AY517596	P. mascareniensis	Vences et al. (2004)	AY517596	NA	NA	NA	NA	NA	NA	Egypt/Kenya
AY517597	P. mascareniensis	Vences et al. (2004)	AY517597	NA	NA	NA	NA	NA	NA	Benin
AY517598	P. mascareniensis	Vences et al. (2004)	AY517598	NA	NA	NA	NA	NA	NA	Ivory Coast
AY517599	P. mascareniensis	Vences et al. (2004)	AY517599	NA	NA	NA	NA	NA	NA	Kenya
AY517600	P. pumilio	Vences et al. (2004)	AY517600	NA	NA	NA	NA	NA	NA	Ghana
AY517601	P. porosissima	Vences et al. (2004)	AY517601	NA	NA	NA	NA	NA	NA	Tanzania
AY517602	P. bibroni	Vences et al. (2004)	AY517602	NA	NA	NA	NA	NA	NA	Ivory Coast
AY517603	P. bibroni	Vences et al. (2004)	AY517603	NA	NA	NA	NA	NA	NA	Ivory Coast
AY517604	P. bibroni	Vences et al. (2004)	AY517604	NA	NA	NA	NA	NA	NA	Gabon
AY517605	P. longirostris	Vences et al. (2004)	AY517605	NA	NA	NA	NA	NA	NA	Ivory Coast
AY517606	P. longirostris	Vences et al. (2004)	AY517606	NA	NA	NA	NA	NA	NA	Ivory Coast
AY517607	P. schubotzi	Vences et al. (2004)	AY517607	NA	NA	NA	NA	NA	NA	Ivory Coast
AY517608	P. schubotzi	Vences et al. (2004)	AY517608	NA	NA	NA	NA	NA	NA	Kenya
AY517609	P. anchietae	Vences et al. (2004)	AY517609	NA	NA	NA	NA	NA	NA	Kenya

AY517610	P. anchietae	Vences et al. (2004)	AY517610	NA	NA	NA	NA	NA	NA	Tanzania
AY517611	P. anchietae	Vences et al. (2004)	AY517611	NA	NA	NA	NA	NA	NA	Kenya
AY517612	P. anchietae	Vences et al. (2004)	AY517612	NA	NA	NA	NA	NA	NA	Kenya
AY517613	P. aequiplicata	Vences et al. (2004)	AY517613	NA	NA	NA	NA	NA	NA	Benin
AY517614	P. aequiplicata	Vences et al. (2004)	AY517614	NA	NA	NA	NA	NA	NA	Ivory Coast
AY517615	P. aequiplicata	Vences et al. (2004)	AY517615	NA	NA	NA	NA	NA	NA	Ivory Coast
AY517616	P. aequiplicata	Vences et al. (2004)	AY517616	NA	NA	NA	NA	NA	NA	Ivory Coast
AY517617	P. aequiplicata	Vences et al. (2004)	AY517617	NA	NA	NA	NA	NA	NA	Ivory Coast
AY517618	P. aequiplicata	Vences et al. (2004)	AY517618	NA	NA	NA	NA	NA	NA	Ivory Coast
AY880532	P. mascareniensis	Delorme, M. (2004)	AY880532	NA	NA	NA	NA	NA	NA	NA
AY948742	P. sp.	Roelants et al. (2007)	AY948742	NA	NA	NA	NA	NA	NA	NA
DQ071575	P. taenioscelis	Bwong, B.A. et al. (Unpublished)	DQ071575	NA	NA	NA	NA	NA	NA	Kenya
DQ347306	P. sp. A	Bossuyt et al. (2006)	DQ347306	NA	NA	NA	NA	NA	NA	NA
DQ347307	P. sp. A	Bossuyt et al. (2006)	DQ347307	NA	NA	NA	NA	NA	NA	NA
DQ525918	P. mahnerti	Measey et al. (2007)	DQ525918	NA	NA	NA	NA	NA	NA	Kenya
DQ525919	P. aff. Aequiplicata	Measey et al. (2007)	DQ525919	NA	NA	NA	NA	NA	NA	Cameroon
DQ525920	P. anchietae	Measey et al. (2007)	DQ525920	NA	NA	NA	NA	NA	NA	Kenya
DQ525921	P. anchietae	Measey et al. (2007)	DQ525921	NA	NA	NA	NA	NA	NA	Somalia
DQ525922	P. nilotica	Measey et al. (2007)	DQ525922	NA	NA	NA	NA	NA	NA	Somalia
DQ525923	P. nilotica	Measey et al. (2007)	DQ525923	NA	NA	NA	NA	NA	NA	Uganda
DQ525924	P. nilotica	Measey et al. (2007)	DQ525924	NA	NA	NA	NA	NA	NA	Kenya
DQ525925	P. nilotica	Measey et al. (2007)	DQ525925	NA	NA	NA	NA	NA	NA	Kenya
DQ525926	P. nilotica	Measey et al. (2007)	DQ525926	NA	NA	NA	NA	NA	NA	Kenya
DQ525927	P. nilotica	Measey et al. (2007)	DQ525927	NA	NA	NA	NA	NA	NA	Kenya
DQ525928	P. nilotica	Measey et al. (2007)	DQ525928	NA	NA	NA	NA	NA	NA	Kenya
DQ525929	P. nilotica	Measey et al. (2007)	DQ525929	NA	NA	NA	NA	NA	NA	Tanzania
DQ525930	P. mascareniensis C	Measey et al. (2007)	DQ525930	NA	NA	NA	NA	NA	NA	Guinea
DQ525931	P. mascareniensis D	Measey et al. (2007)	DQ525931	NA	NA	NA	NA	NA	NA	Uganda
DQ525932	P. mascareniensis E	Measey et al. (2007)	DQ525932	NA	NA	NA	NA	NA	NA	Central African Republic

DQ525933	P. newtoni	Measey et al. (2007)	DQ525933	NA	NA	NA	NA	NA	NA		São Tomé
DQ525934	P. newtoni	Measey et al. (2007)	DQ525934	NA	NA	NA	NA	NA	NA		São Tomé
DQ525935	P. newtoni	Measey et al. (2007)	DQ525935	NA	NA	NA	NA	NA	NA		São Tomé
DQ525936	P. newtoni	Measey et al. (2007)	DQ525936	NA	NA	NA	NA	NA	NA		São Tomé
DQ525937	P. newtoni	Measey et al. (2007)	DQ525937	NA	NA	NA	NA	NA	NA		São Tomé
DQ525938	P. newtoni	Measey et al. (2007)	DQ525938	NA	NA	NA	NA	NA	NA		São Tomé
DQ525939	P. oxyrhynchus	Measey et al. (2007)	DQ525939	NA	NA	NA	NA	NA	NA		Malawi
DQ525940	P. porosissima A	Measey et al. (2007)	DQ525940	NA	NA	NA	NA	NA	NA		Tanzania
DQ525941	P. porosissima	Measey et al. (2007)	DQ525941	NA	NA	NA	NA	NA	NA		Tanzania
DQ525942	P. pumilio	Measey et al. (2007)	DQ525942	NA	NA	NA	NA	NA	NA		Guinea
DQ525943	P. taenioscelis	Measey et al. (2007)	DQ525943	NA	NA	NA	NA	NA	NA		Kenya
DQ525944	P. sp.	Measey et al. (2007)	DQ525944	NA	NA	NA	NA	NA	NA		Tanzania
DQ525945	P. uzungwensis	Measey et al. (2007)	DQ525945	NA	NA	NA	NA	NA	NA		Tanzania
GQ183592	P. mascareniensis	Siow, M.T.M. (Unpublished)	GQ183592	NA	NA	NA	NA	NA	NA		Uganda
GQ183593	P. mascareniensis	Siow, M.T.M. (Unpublished)	GQ183593	NA	NA	NA	NA	NA	NA		Uganda
GQ183594	P. mascareniensis	Siow, M.T.M. (Unpublished)	GQ183594	NA	NA	NA	NA	NA	NA		Uganda
GQ183595	P. christyi	Siow, M.T.M. (Unpublished)	GQ183595	NA	NA	NA	NA	NA	NA		Uganda
GQ183596	P. anchietae	Siow, M.T.M. (Unpublished)	GQ183596	NA	NA	NA	NA	NA	NA		Uganda
GQ183597	P. anchietae	Siow, M.T.M. (Unpublished)	GQ183597	NA	NA	NA	NA	NA	NA		Uganda
GQ183598	P. anchietae	Siow, M.T.M. (Unpublished)	GQ183598	NA	NA	NA	NA	NA	NA		Uganda
GU457590	P. newtoni	Zimkus et al. (2010)	GU457590	NA	NA	NA	NA	NA	NA		Sao Tome
GU457591	P. newtoni	Zimkus et al. (2010)	GU457591	NA	NA	NA	NA	NA	NA		Sao Tome
HM364770	P. mascareniensis	Rosa, G.M. et al. (Unpublished)	HM364770	NA	NA	NA	NA	NA	NA		Madagascar
JF903871	P. mascareniensis	Crottini, A. et al. (Unpublished)	JF903871	NA	NA	NA	NA	NA	NA		Madagascar
JF903872	P. mascareniensis	Crottini, A. et al. (Unpublished)	JF903872	NA	NA	NA	NA	NA	NA		Madagascar
JX101747	P. mascareniensis	Welton, C. et al. (Unpublished)	JX101747	NA	NA	NA	NA	NA	NA		Madagascar
JX101748	P. mascareniensis	Welton, C. et al. (Unpublished)	JX101748	NA	NA	NA	NA	NA	NA		Madagascar

JX464828	P. nana	Mengistu, A.A. (2012)	JX464828	NA	NA	NA	NA	NA	NA	Ethiopia
JX464829	P. nana	Mengistu, A.A. (2012)	JX464829	NA	NA	NA	NA	NA	NA	Ethiopia
JX464830	P. nana	Mengistu, A.A. (2012)	JX464830	NA	NA	NA	NA	NA	NA	Ethiopia
JX464831	P. sp.	Mengistu, A.A. (2012)	JX464831	NA	NA	NA	NA	NA	NA	Ethiopia
JX464832	P. nana	Mengistu, A.A. (2012)	JX464832	NA	NA	NA	NA	NA	NA	Ethiopia
JX464833	P. nana	Mengistu, A.A. (2012)	JX464833	NA	NA	NA	NA	NA	NA	Ethiopia
JX464834	P. nana	Mengistu, A.A. (2012)	JX464834	NA	NA	NA	NA	NA	NA	Ethiopia
JX464835	P. nana	Mengistu, A.A. (2012)	JX464835	NA	NA	NA	NA	NA	NA	Ethiopia
JX464836	P. nana	Mengistu, A.A. (2012)	JX464836	NA	NA	NA	NA	NA	NA	Ethiopia
JX464837	P. nana	Mengistu, A.A. (2012)	JX464837	NA	NA	NA	NA	NA	NA	Ethiopia
JX464838	P. cooperi	Mengistu, A.A. (2012)	JX464838	NA	NA	NA	NA	NA	NA	Ethiopia
JX464839	P. cooperi	Mengistu, A.A. (2012)	JX464839	NA	NA	NA	NA	NA	NA	Ethiopia
JX464840	P. sp.	Mengistu, A.A. (2012)	JX464840	NA	NA	NA	NA	NA	NA	Ethiopia
JX464841	P. sp.	Mengistu, A.A. (2012)	JX464841	NA	NA	NA	NA	NA	NA	Ethiopia
JX464842	P. erlangeri	Mengistu, A.A. (2012)	JX464842	NA	NA	NA	NA	NA	NA	Ethiopia
JX464843	P. sp.	Mengistu, A.A. (2012)	JX464843	NA	NA	NA	NA	NA	NA	Ethiopia
JX464844	P. neumanni	Mengistu, A.A. (2012)	JX464844	NA	NA	NA	NA	NA	NA	Ethiopia
JX464845	P. sp.	Mengistu, A.A. (2012)	JX464845	NA	NA	NA	NA	NA	NA	Ethiopia
JX464846	P. sp.	Mengistu, A.A. (2012)	JX464846	NA	NA	NA	NA	NA	NA	Ethiopia
JX464847	P. neumanni	Mengistu, A.A. (2012)	JX464847	NA	NA	NA	NA	NA	NA	Ethiopia
JX464848	P. neumanni	Mengistu, A.A. (2012)	JX464848	NA	NA	NA	NA	NA	NA	Ethiopia
JX464849	P. neumanni	Mengistu, A.A. (2012)	JX464849	NA	NA	NA	NA	NA	NA	Ethiopia
JX464850	P. neumanni	Mengistu, A.A. (2012)	JX464850	NA	NA	NA	NA	NA	NA	Ethiopia
JX464851	P. neumanni	Mengistu, A.A. (2012)	JX464851	NA	NA	NA	NA	NA	NA	Ethiopia
JX464852	P. neumanni	Mengistu, A.A. (2012)	JX464852	NA	NA	NA	NA	NA	NA	Ethiopia
JX464853	P. anchietae	Mengistu, A.A. (2012)	JX464853	NA	NA	NA	NA	NA	NA	Ethiopia
JX464854	P. neumanni	Mengistu, A.A. (2012)	JX464854	NA	NA	NA	NA	NA	NA	Ethiopia

JX464855	P. neumanni	Mengistu, A.A. (2012)	JX464855	NA	NA	NA	NA	NA	NA	Ethiopia
JX464856	P. neumanni	Mengistu, A.A. (2012)	JX464856	NA	NA	NA	NA	NA	NA	Ethiopia
JX464857	P. neumanni	Mengistu, A.A. (2012)	JX464857	NA	NA	NA	NA	NA	NA	Ethiopia
JX464858	P. neumanni	Mengistu, A.A. (2012)	JX464858	NA	NA	NA	NA	NA	NA	Ethiopia
JX464859	P. neumanni	Mengistu, A.A. (2012)	JX464859	NA	NA	NA	NA	NA	NA	Ethiopia
JX464860	P. neumanni	Mengistu, A.A. (2012)	JX464860	NA	NA	NA	NA	NA	NA	Ethiopia
JX464861	P. neumanni	Mengistu, A.A. (2012)	JX464861	NA	NA	NA	NA	NA	NA	Ethiopia
JX464862	P. neumanni	Mengistu, A.A. (2012)	JX464862	NA	NA	NA	NA	NA	NA	Ethiopia
JX464863	P. neumanni	Mengistu, A.A. (2012)	JX464863	NA	NA	NA	NA	NA	NA	Ethiopia
JX464864	P. neumanni	Mengistu, A.A. (2012)	JX464864	NA	NA	NA	NA	NA	NA	Ethiopia
JX464865	P. neumanni	Mengistu, A.A. (2012)	JX464865	NA	NA	NA	NA	NA	NA	Ethiopia
JX464866	P. neumanni	Mengistu, A.A. (2012)	JX464866	NA	NA	NA	NA	NA	NA	Ethiopia
JX464867	P. sp.	Mengistu, A.A. (2012)	JX464867	NA	NA	NA	NA	NA	NA	Ethiopia
JX464868	P. nana	Mengistu, A.A. (2012)	JX464868	NA	NA	NA	NA	NA	NA	Ethiopia
JX464869	P. nana	Mengistu, A.A. (2012)	JX464869	NA	NA	NA	NA	NA	NA	Ethiopia
JX464870	P. nana	Mengistu, A.A. (2012)	JX464870	NA	NA	NA	NA	NA	NA	Ethiopia
JX464871	P. erlangeri	Mengistu, A.A. (2012)	JX464871	NA	NA	NA	NA	NA	NA	Ethiopia
JX464872	P. wadei	Mengistu, A.A. (2012)	JX464872	NA	NA	NA	NA	NA	NA	Ethiopia
JX464873	P. wadei	Mengistu, A.A. (2012)	JX464873	NA	NA	NA	NA	NA	NA	Ethiopia
JX464874	P. sp.	Mengistu, A.A. (2012)	JX464874	NA	NA	NA	NA	NA	NA	Ethiopia
JX464875	P. sp.	Mengistu, A.A. (2012)	JX464875	NA	NA	NA	NA	NA	NA	Ethiopia
JX464876	P. sp.	Mengistu, A.A. (2012)	JX464876	NA	NA	NA	NA	NA	NA	Ethiopia
JX464877	P. sp.	Mengistu, A.A. (2012)	JX464877	NA	NA	NA	NA	NA	NA	Ethiopia
JX464878	P. anchietae	Mengistu, A.A. (2012)	JX464878	NA	NA	NA	NA	NA	NA	Ethiopia
JX464879	P. anchietae	Mengistu, A.A. (2012)	JX464879	NA	NA	NA	NA	NA	NA	Ethiopia
JX464880	P. mascareniensis	Mengistu, A.A. (2012)	JX464880	NA	NA	NA	NA	NA	NA	Ethiopia
JX464881	P. anchietae	Mengistu, A.A. (2012)	JX464881	NA	NA	NA	NA	NA	NA	Ethiopia

JX464882	P. anchietae	Mengistu, A.A. (2012)	JX464882	NA	NA	NA	NA	NA	NA	Ethiopia
JX464883	P. mascareniensis	Mengistu, A.A. (2012)	JX464883	NA	NA	NA	NA	NA	NA	Ethiopia
JX464884	P. mascareniensis	Mengistu, A.A. (2012)	JX464884	NA	NA	NA	NA	NA	NA	Ethiopia
JX464885	P. mascareniensis	Mengistu, A.A. (2012)	JX464885	NA	NA	NA	NA	NA	NA	Ethiopia
JX464886	P. mascareniensis	Mengistu, A.A. (2012)	JX464886	NA	NA	NA	NA	NA	NA	Ethiopia
JX464887	P. mascareniensis	Mengistu, A.A. (2012)	JX464887	NA	NA	NA	NA	NA	NA	Ethiopia
JX464888	P. nana	Mengistu, A.A. (2012)	JX464888	NA	NA	NA	NA	NA	NA	Ethiopia
JX464889	P. nana	Mengistu, A.A. (2012)	JX464889	NA	NA	NA	NA	NA	NA	Ethiopia
JX464890	P. nana	Mengistu, A.A. (2012)	JX464890	NA	NA	NA	NA	NA	NA	Ethiopia
JX464891	P. cooperi	Mengistu, A.A. (2012)	JX464891	NA	NA	NA	NA	NA	NA	Ethiopia
JX464892	P. cooperi	Mengistu, A.A. (2012)	JX464892	NA	NA	NA	NA	NA	NA	Ethiopia
JX464893	P. sp.	Mengistu, A.A. (2012)	JX464893	NA	NA	NA	NA	NA	NA	Ethiopia
JX464894	P. sp.	Mengistu, A.A. (2012)	JX464894	NA	NA	NA	NA	NA	NA	Ethiopia
JX464895	P. sp.	Mengistu, A.A. (2012)	JX464895	NA	NA	NA	NA	NA	NA	Ethiopia
JX464896	P. sp.	Mengistu, A.A. (2012)	JX464896	NA	NA	NA	NA	NA	NA	Ethiopia
JX464897	P. sp.	Mengistu, A.A. (2012)	JX464897	NA	NA	NA	NA	NA	NA	Ethiopia
JX464898	P. sp.	Mengistu, A.A. (2012)	JX464898	NA	NA	NA	NA	NA	NA	Ethiopia
KC179964	P. anchietae	de Sá, R.O. et al. (2012)	KC179964	NA	NA	NA	NA	NA	NA	Tanzania
KF027211	P. nilotica	Dehling, J.M. and Sinsch, U. (2013)	KF027211	NA	NA	NA	NA	NA	NA	Rwanda
KF027212	P. porosissima	Dehling, J.M. and Sinsch, U. (2013)	KF027212	NA	NA	NA	NA	NA	NA	Rwanda
KF027213	P. anchietae	Dehling, J.M. and Sinsch, U. (2013)	KF027213	NA	NA	NA	NA	NA	NA	Rwanda
KF027214	P. nilotica	Dehling, J.M. and Sinsch, U. (2013)	KF027214	NA	NA	NA	NA	NA	NA	Rwanda
KF178892	P. perreti	Zimkus, B.M. and Larson, J.G. (2013)	KF178892	NA	NA	NA	NA	NA	NA	Gabon

KF178893	P. taenioscelis	Zimkus, B.M. and Larson, J.G. (2013)	KF178893	NA	NA	NA	NA	NA	NA	Gabon
KF178894	P. uzungwensis	Zimkus, B.M. and Larson, J.G. (2013)	KF178894	NA	NA	NA	NA	NA	NA	Gabon
KF410853	P. uzungwensis	Mueller, H. and Larson, J. (2013)	KF410853	NA	NA	NA	NA	NA	NA	Tanzania
KF410854	P. uzungwensis	Mueller, H. and Larson, J. (2013)	KF410854	NA	NA	NA	NA	NA	NA	Tanzania
KF991275	P. aequiplicata	Barej, M.F. et al. (2014)	KF991275	NA	NA	NA	NA	NA	NA	Liberia
KJ541761	P. chrysogaster	Dehling, J.M. and Sinsch, U. (2013)	KJ541761	NA	NA	NA	NA	NA	NA	Rwanda
Dyscophus_guineti	Dyscophus guineti	Vences et al. (2000)	AF124122	AF124099	NA	NA	NA	NA	NA	NA
Dyscophus_guineti	Dyscophus guineti	Kurabayashi, A. et al. (2011)	NA	NA	AB611894	AB611895	AB611899	AB611891	NA	NA
Micrixalus_fuscus	Micrixalus fuscus	Meenakshi, K. et al. (2009)	GUI136106	GUI143817	NA	NA	NA	NA	NA	NA
Micrixalus_fuscus	Micrixalus fuscus	Barej, M.F. et al. (2014)	NA	NA	NA	NA	K991333	NA	NA	India
Micrixalus_fuscus	Micrixalus fuscus	Bossuyt and Milinkovitch, M.C. (2000)	NA	NA	NA	NA	NA	AF249183	NA	NA

Table S2: Primers used in this study

Gene	Primer Name	Primer Sequence	Annealing Temp (T_a)	Source
12S	12SAL	AAACTGGGATTAGATACCCCACTAT	57°C	Kocher *et al*. (1989)/ Hrbek and Larson (1999)
	12SBH	GAGGGTGACGGGCGGTGTGT		
16S	16SAL	CGCCTGTTTAYCAAAAACAT	54°C	Kessing *et al*. (1989)
	16SBR	CCGGTYTGAACTCAGATCAYGT		
Rag-1	Rag1-PtyF	CCGTTCTGTTGATGAATACCC	48-53°C	Freilich *et al*. (2014)
	Rag-1-PtyR	TAAGGGTTGGCTCTCCATGT		
NCX1	NCX1-PtyF	CCAAATGGTGAAACCACAAA	51-56°C	Freilich *et al*. (2014)
	NCX1-PtyR	ACCTCCTCGACGTACAATGG		
	NacaA	TTTGTTGCCATGGTTTACATGTT	51-56°C	Roelants and Bossuyt (2005)
	NacaO	ATACCTGCATGATCATCATCAAA		
Tyrosinase	TyrC-F	GGCAGAGGAWCRTGCCAAGATGT	55-60°C	Bossuyt and Milinkovitch (2000)
	TyrG-R	TGCTGGCRTCTCTCCARTCCCA		
CXCR4	CXCR4-PtyF	ATCCAAGCAAACCAAAGGTG	56°C	Freilich *et al*. (2014)
	CXCR4-PtyR	CCGTCCATGTCATCTATACGG		

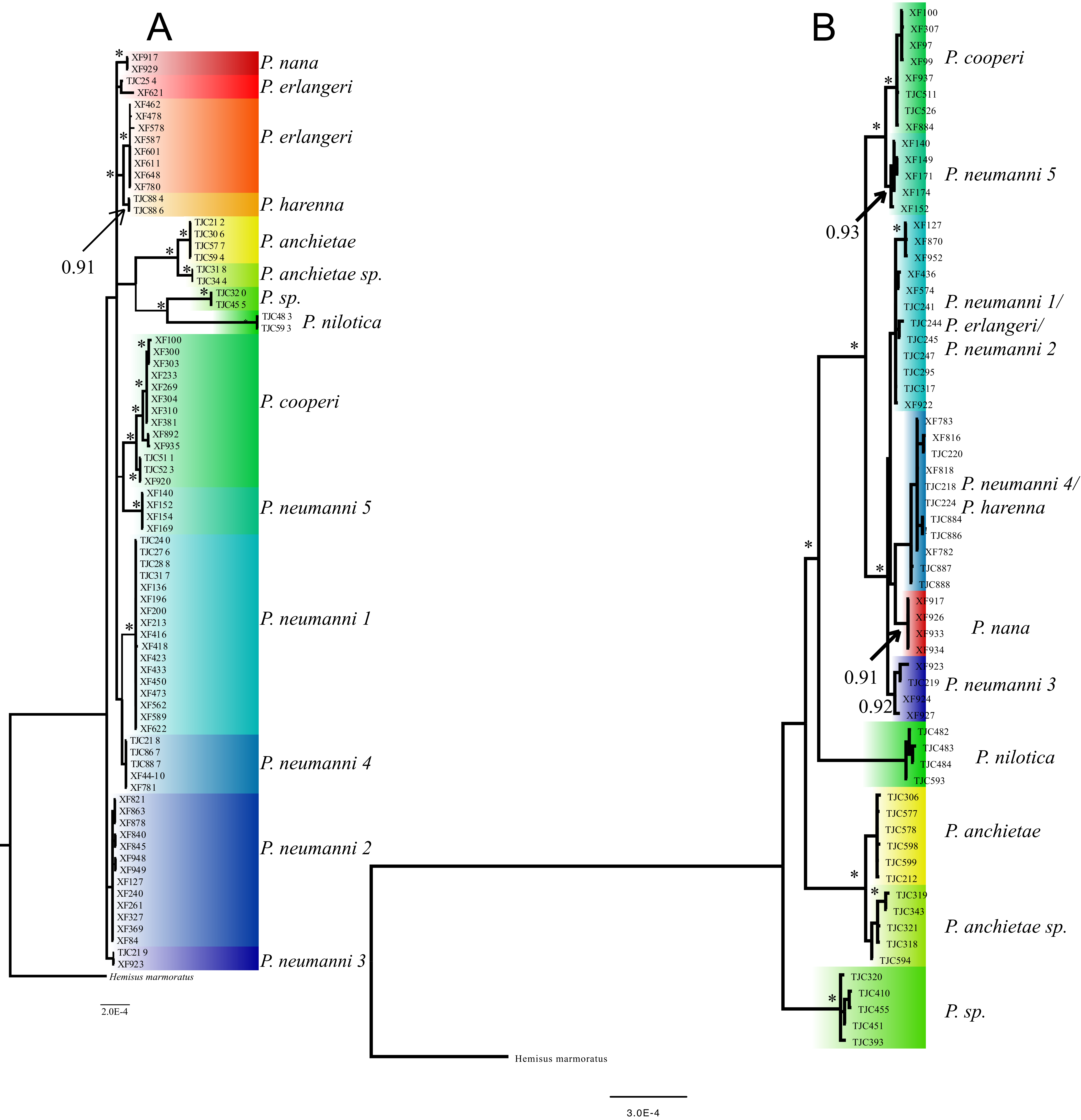

Figure S1: Bayesian phylogeny of Ethiopian *Ptychadena* from this study based on concatenated alignments of the A) 12S and 16S mt loc[illegible] B) all four nuclear genes analyzed in this study. Clades are indicated with highlighting. Posterior probabilities greater than 0.95 are indicated with asterisks.

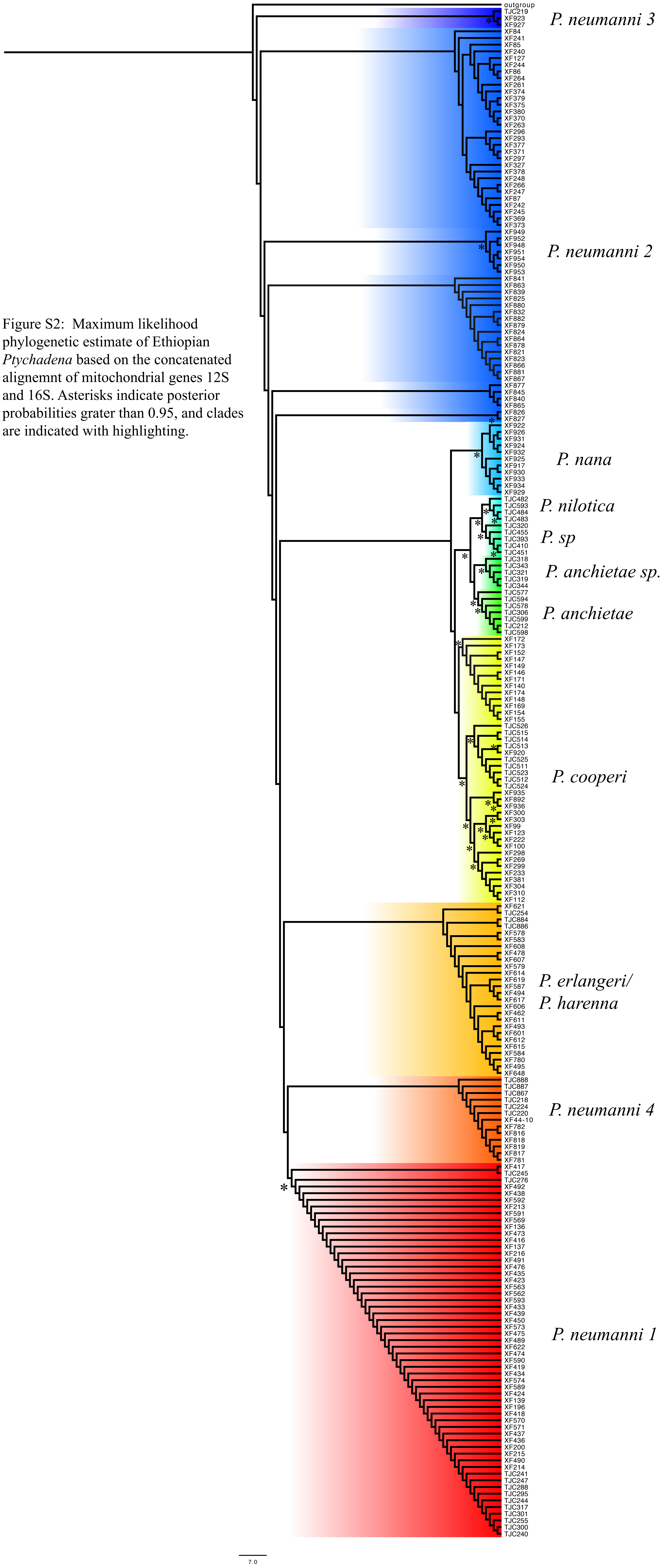

Figure S2: Maximum likelihood phylogenetic estimate of Ethiopian *Ptychadena* based on the concatenated alignemnt of mitochondrial genes 12S and 16S. Asterisks indicate posterior probabilities grater than 0.95, and clades are indicated with highlighting.

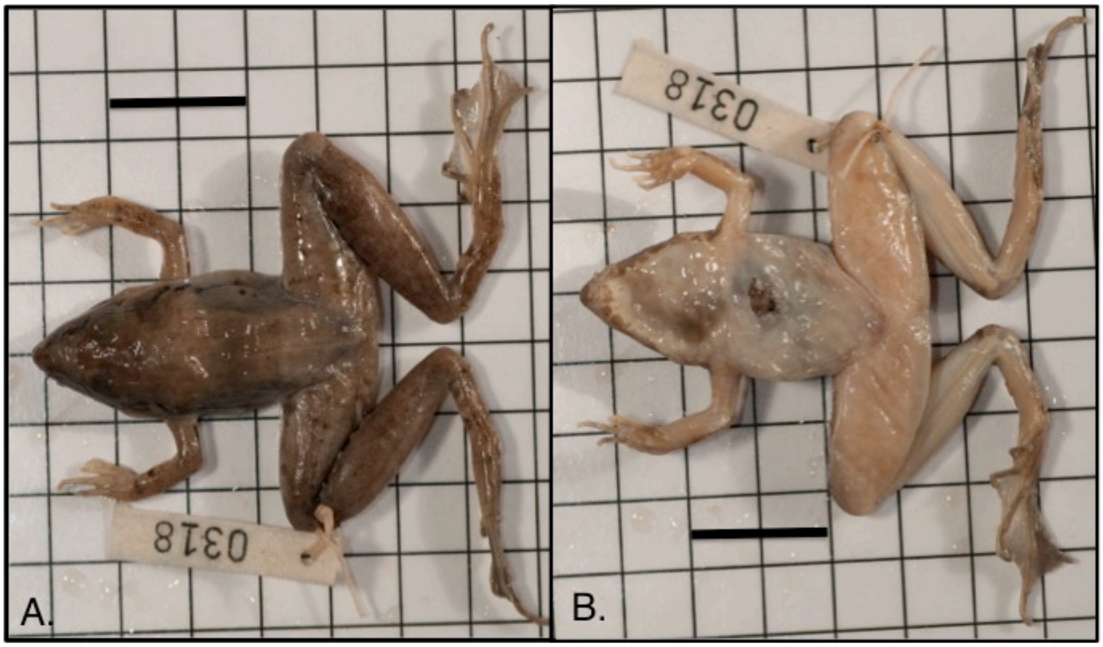

Figure S3. Dorsal (A.) and ventral (B.) views of the holotype of *Ptychadena baroensis* **sp. nov**. Inset scale bars = 2cm.

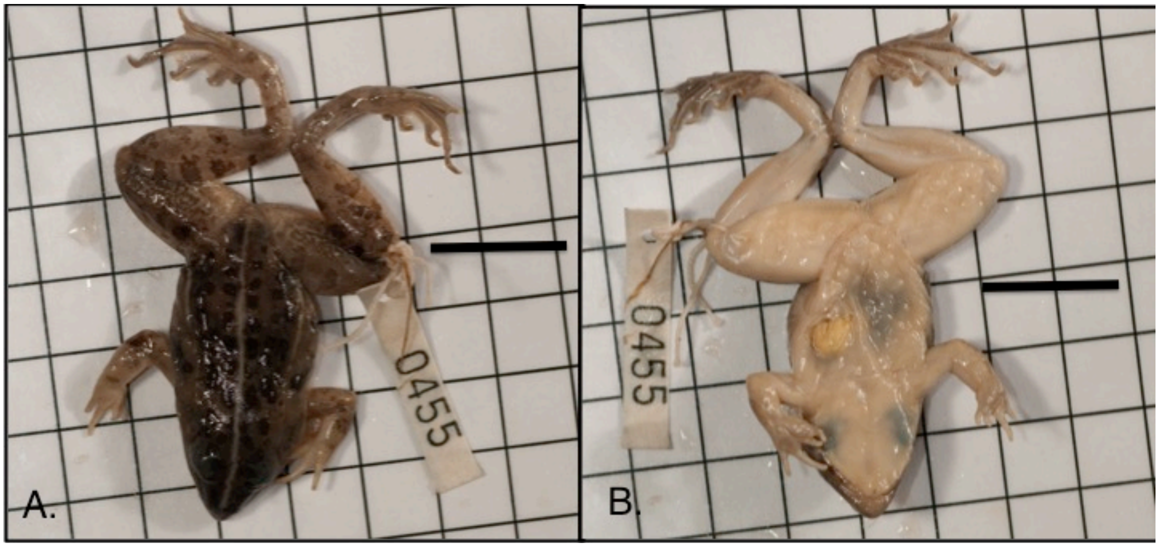

Figure S4. Dorsal (A.) and ventral (B.) views of the holotype of *Ptychadena neurensis* **sp. nov**. Inset scale bars = 2cm.

Information on PCR Conditions

We used 13-μL PCR reactions. For the mitochondrial genes 12S and 16S and nuclear genes Tyrosinase and CXCR4 there was an initial denaturation step for 30 seconds at 98°C followed by 40 cycles for 5 seconds at 98°C, 10 seconds at a gradient of annealing temperatures (Table S2), and 1 minute at 72°C, followed by a 5 minute extension at 72°C. For nuclear gene Rag-1 there was an initial denaturation step for 30 seconds at 98°C, followed by 25 cycles of 5 seconds at 98°C, 5 seconds at a gradient of annealing temperatures (Table S2), and 45 seconds at 72°C, followed by a 10 minute extension at 72°C. For nuclear gene NCX1 there was an initial denaturation step for 1 minute at 98°C, followed by 30 cycles of 20 seconds at 98°C, 20 seconds at a gradient of annealing temperatures (Table S2), and 40 seconds at 72°C, followed by a 10 minute extension at 72°C. All PCR products were purified using ExoSap-IT (USB Corp.).

Gene Tree Analyses—Methods

To estimate the relationships among Ethiopian highland and widespread lowland *Ptychadena,* phylogenies were reconstructed using both Bayesian (BI) and maximum likelihood (ML) methods. For all genes, ML phylogenies were reconstructed using RAxML v7.0.4 on XSEDE (Stamatakis 2014), and the support for nodes was assessed via 1,000 bootstrap replicates. Bayesian analyses of relationships were run for a concatenated dataset of the two mitochondrial loci and for a concatenated dataset of the four nuclear loci using MrBayes 3.2.6 on XSEDE (Ronquist *et al.* 2012). Analyses were run for 50,000,000 generations over four chains. We sampled 50,000 trees and discarded the first 12,500 as burn-in based on stationarity of the log-likelihood scores.

Gene Tree Analyses—Results

Our Bayesian analysis of the concatenated mitochondrial (mt) dataset recovers seven of the eight highland lineages suggested by Freilich *et al*. (2014, Figure S1A), with strong support for four of these—*P. nana, P. cooperi, P. neumanni 5,* and *P. neumanni 1*. The addition of samples from *P. harenna* resulted in the failure of our phylogeny to recover *P. erlangeri* as a monophyletic clade. The mt phylogeny weakly supports the monophyly of the previously unsampled rare species *P. harenna* (posterior probability (pp) = 0.91), which is strongly supported as a sister lineage to one mtDNA clade of *P. erlangeri*. Additionally, the mt phylogeny supports the existence of two geographically distinct clades of both of the widespread lowland species. The ML phylogeny reconstructed in this study based on the concatenated mt dataset (Figure S2) differs from the Bayesian phylogeny (Figure S1A) primarily in that it strongly supports the monophyly of *P. neumanni 3* and fails to recover a monophyletic *P. harenna.*

The Bayesian phylogeny based on the concatenated nuclear loci (Figure S1B) differs from the mt phylogenies in several ways: 1) it fails to support the monophyly of *P. neumanni 1 and P. harenna* and weakly supports the monophyly of *P. neumanni 3* (pp= 0.92), P. neumanni *5* (pp = 0.93), and *P. nana* (pp = 0.91); 2) it strongly supports the monophyly of the clade containing *P. neumanni 5* and *P. cooperi*; 3) it strongly supports the monophyly of the highland lineages; and 4) it fails to recovery the monophyly of the two lineages morphologically identified as *P. mascareniensis* (*P. nilotica* and *P. sp.* in Figure 3). Additionally, two *P. nana* samples group with *P.*

neumanni 3 in the nuclear phylogeny, and one *P. anchietae* sample that groups with *P. anchietae* in the mt phylogeny groups with *P. anchietae* sp. in the nuclear phylogeny. The ML phylogeny based on the nuclear genes does not differ substantially from the Bayesian phylogeny.

References

Barej MF, Schmitz A, Günther R *ym.* (2014) The first endemic West African vertebrate family–a new anuran family highlighting the uniqueness of the Upper Guinean biodiversity hotspot. *Frontiers in zoology*, **11**, 8.

Bossuyt F, Brown RM, Hillis DM, Cannatella DC, Milinkovitch MC (2006) Phylogeny and biogeography of a cosmopolitan frog radiation: Late cretaceous diversification resulted in continent-scale endemism in the family ranidae. *Systematic biology*, **55**, 579–594.

Bossuyt F, Milinkovitch MC (2000) Convergent adaptive radiations in Madagascan and Asian ranid frogs reveal covariation between larval and adult traits. *Proceedings of the National Academy of Sciences*, **97**, 6585–6590.

Dehling JM, Sinsch U (2013) Diversity of Ptychadena in Rwanda and taxonomic status of P. chrysogaster Laurent, 1954 (Amphibia, Anura, Ptychadenidae). *ZooKeys*, 69.

Delorme, M. (2004) Phylogeny of Ranidae Rhacophorinae: Conflicts between molecular and morphological analyses, and study of its nature. *Thesis, Museum National d'Histoire Naturelle, Paris, France*.

Freilich X, Tollis M, Boissinot S (2014) Hiding in the highlands: Evolution of a frog species complex of the genus Ptychadena in the Ethiopian highlands. *Molecular Phylogenetics and Evolution*, **71**, 157–169.

Hrbek T, Larson a (1999) The evolution of diapause in the killifish family Rivulidae (Atherinomorpha, Cyprinodontiformes): a molecular phylogenetic and biogeographic perspective. *Evolution*, **53**, 1200–1216.

John Measey G, Vences M, Drewes RC *ym.* (2007) Freshwater paths across the ocean: molecular phylogeny of the frog Ptychadena newtoni gives insights into amphibian colonization of oceanic islands. *Journal of Biogeography*, **34**, 7–20.

Kessing B, Croom H, Martin A *ym.* (1989) The simple fool's guide to PCR. *University of Hawaii, Honolulu*.

Kocher TD, Thomas WK, Meyer a *ym.* (1989) Dynamics of mitochondrial DNA evolution in animals: amplification and sequencing with conserved primers. *Proceedings of the National Academy of Sciences of the United States of America*, **86**, 6196–6200.

Kurabayashi A, Matsui M, Belabut DM *ym.* (2011) From Antarctica or Asia? New colonization scenario for Australian-New Guinean narrow mouth toads suggested from the findings on a mysterious genus Gastrophrynoides. *BMC evolutionary biology*, **11**, 175.

Meenakshi K, Sujith VG, Sanil G (2009) DNA barcoding of some amphibians of Western Ghats. In: *Third International Barcode of Life Conference*

Mengistu AA (2012) Amphibian diversity, distribution and conservation in the Ethiopian highlands: morphological, molecular and biogeographic investigation on Leptopelis and Ptychadena (Anura).

Müller H, Larson JG (2014) Description of the Tadpole of Ptychadena uzungwensis (Amphibia: Anura: Ptychadenidae) from the Southern Udzungwa Mountains, Tanzania. *Journal of Herpetology*, **48**, 284–290.

Roelants K, Bossuyt F (2005) Archaeobatrachian paraphyly and Pangaean diversification of crown-group frogs. *Systematic Biology*, **54**, 111–126.

Roelants K, Gower DJ, Wilkinson M *ym.* (2007) Global patterns of diversification in the history of modern amphibians. *Proceedings of the National Academy of Sciences*, **104**, 887–892.

Ronquist F, Teslenko M, Van Der Mark P *ym.* (2012) Mrbayes 3.2: Efficient bayesian phylogenetic inference and model choice across a large model space. *Systematic Biology*,

61, 539–542.
de Sá RO, Streicher JW, Sekonyela R *ym.* (2012) Molecular phylogeny of microhylid frogs (Anura: Microhylidae) with emphasis on relationships among New World genera. *BMC Evolutionary Biology*, **12**, 241.
Stamatakis A (2014) RAxML version 8: A tool for phylogenetic analysis and post-analysis of large phylogenies. *Bioinformatics*, **30**, 1312–1313.
Vences M (1999) *Phylogenetic Studies on Ranoid Frogs (Amphibia: Anura): With a Discussion of the Origin and Evolution of the Vertebrate Clades of Madagascar*. Rheinische Friedrich-Wilhelms-Universität zu Bonn.
Vences M, Kosuch J, Rödel M *ym.* (2004) Phylogeography of Ptychadena mascareniensis suggests transoceanic dispersal in a widespread African-Malagasy frog lineage. *Journal of Biogeography*, **31**, 593–601.
Zimkus BM, Larson JG (2013) Assessment of the amphibians of Batéké Plateau National Park, Gabon, including results of chytrid pathogen tests. *Salamandra*, **49**, 159–170.
Zimkus BM, Rödel M-O, Hillers A (2010) Complex patterns of continental speciation: molecular phylogenetics and biogeography of sub-Saharan puddle frogs (Phrynobatrachus). *Molecular Phylogenetics and Evolution*, **55**, 883–900.
